# Climatic Correlates of Tree Mortality in Water- and Energy-Limited Forests

**DOI:** 10.1371/journal.pone.0069917

**Published:** 2013-07-25

**Authors:** Adrian J. Das, Nathan L. Stephenson, Alan Flint, Tapash Das, Phillip J. van Mantgem

**Affiliations:** 1 Western Ecological Research Center, United States Geological Survey, Three Rivers, California, United States of America; 2 California Water Science Center, United States Geological Survey, Sacramento, California, United States of America; 3 Climate Atmospheric Science and Physical Oceanography, Scripps Institution of Oceanography, La Jolla, California, United States of America; 4 Western Ecological Research Center, United States Geological Survey, Arcata, California, United States of America; The Ohio State University, United States of America

## Abstract

Recent increases in tree mortality rates across the western USA are correlated with increasing temperatures, but mechanisms remain unresolved. Specifically, increasing mortality could predominantly be a consequence of temperature-induced increases in either (1) drought stress, or (2) the effectiveness of tree-killing insects and pathogens. Using long-term data from California’s Sierra Nevada mountain range, we found that in water-limited (low-elevation) forests mortality was unambiguously best modeled by climatic water deficit, consistent with the first mechanism. In energy-limited (high-elevation) forests deficit models were only equivocally better than temperature models, suggesting that the second mechanism is increasingly important in these forests. We could not distinguish between models predicting mortality using absolute versus relative changes in water deficit, and these two model types led to different forecasts of mortality vulnerability under future climate scenarios. Our results provide evidence for differing climatic controls of tree mortality in water- and energy-limited forests, while highlighting the need for an improved understanding of tree mortality processes.

## Introduction

Recent regional increases in tree mortality rates and episodes of forest die-back have been linked to rising temperatures [1], [2], indicating that a potentially substantial source of biotic feedbacks to global climatic changes may already be underway [3]. Assessing the effect of such changes will require a realistic understanding of the relationships between climate and tree mortality as well as the mechanisms that underlie those relationships.

For example, increases in tree mortality with temperature might be attributed to two broad and non-mutually exclusive mechanisms: (i) increasing drought stress on trees resulting from temperature-induced increases in climatic water deficit (hereafter “deficit” : an index of evaporative demand that is not met by available water, hence drought stress [4], [5]) [2], [6]–[9], or (ii) temperature-induced increases in the reproduction, survivorship, and effectiveness of insects and pathogens that kill trees [10]–[12]. The first hypothesis posits that changes in mortality rate are most strongly tied to tree condition, with increasing drought stress making a given tree more susceptible to an array of mortality risks including physiological decline and attack by enemies. The second hypothesis posits that changes in mortality rate are most closely tied to the population dynamics and effectiveness of tree enemies, regardless of the condition of their hosts.

We might reasonably hypothesize that the first of these mechanisms dominates in water-limited forests: forests in which growth and other biological processes respond most strongly to changes in water availability, such as in arid regions or, in many mountain ranges, at lower elevations. The second mechanism might be expected to become more important in energy-limited forests: forests in which growth and other biological processes respond most strongly to temperature changes, such as in wet regions or at higher elevations. Although some studies have examined differences in the climatic controls of tree growth rates between water- and energy-limited forests [13], we are unaware of comparable studies of mortality rates.

Even for sites at which drought stress is clearly the best predictor of changes in mortality, the best model for relating changes in mortality to changes in deficit may not be obvious. Trees are adapted to the typical deficit within their geographical range limits, with trees in drier environments being more strongly adapted to lower water availability [14], [15]. In addition, some evidence suggests that trees have some ability to acclimate to changes in water stress, in the short term by altering stomatal conductance and in the longer term by altering carbon allocation priorities or morphological traits [14], [16]–[18].

But how is that range of adaptation defined relative to the average historical deficit at a given site? For example, are species adapted to similar absolute ranges of deficit, regardless of environment? Or are trees that are adapted to drier environments more strongly resistant to changes in deficit than trees adapted to more mesic environments? In short, does one expect mortality to be more strongly correlated with absolute or relative changes in deficit?

Arguments can be made for both possibilities. Trees in more water stressed environments have developed adaptations for survival in those environments (e.g., [8], [14]), perhaps suggesting that they have increased resistance to absolute changes in water stress. Such adaptations might result in mortality having a relative relationship to changes in deficit (i.e., the more water stressed a site is, the stronger the adaptions to absolute increases in water stress). However, in some cases, severe drought results in larger increases in mortality rates at drier sites when compared to more mesic sites [19], [20], perhaps indicating that absolute changes in deficit might be more predictive. While a recent global analysis of the safety margins trees maintain against drought-induced hydraulic failure provides an important first step toward distinguishing among the possibilities [15], alone it does not allow us to definitively choose between them. That work, while showing patterns of embolism resistance that might argue that tree responses to drought are best related to absolute changes in deficit, does not directly relate those safety margins to mortality and also does not address other factors related to hydraulic safety margins such as stomatal regulation.

The precise nature of these relationships (water limitation versus energy limitation, absolute versus relative changes in climatic water deficit) could result in markedly different assessments of the potential vulnerability of a given forest to climate change. For example, many high elevation forests might be substantially more vulnerable if mortality is most strongly related to relative changes in deficit (since many high elevation forests have a relatively small baseline deficit), and forests that experience relatively little change in deficit, due to abundant water and deep soils, might still be substantially vulnerable to climate change if changes in mortality rate are primarily related to changes in climatic favorability to tree enemies.

In this work, we seek to shed light on the possible mechanisms by which increasing temperature can lead to increasing tree mortality rates and to explore some implications of our findings for forecasting changes in tree mortality rates. Specifically, we use empirical data to test.

1. The hypothesis that changes in tree mortality rates in water-limited forests should best correlate (positively) with climatic water deficit, whereas mortality rates in energy-limited forests should best correlate (positively) with temperature due to temperature’s direct effects on insect and pathogen populations (e.g., [21], [22], [23]).
2. Whether absolute or relative changes in deficit best correlate with changes in mortality rate.

With regard to #1, because deficit partly depends on temperature, deficit and temperature are correlated. However, deficit also depends on water availability, which can vary independently of temperature; for example, a warm, wet summer will have a low deficit while a warm, dry summer will have a high deficit. We therefore might reasonably expect to be able to distinguish between deficit- and temperature-related changes in tree mortality rates.

Using results of these analyses, we then explore the implications of our findings by forecasting responses of tree mortality rates to different scenarios of future climatic changes. In particular, we use our models and forecasts to provide additional insights into climatic controls of tree mortality and to highlight critical knowledge gaps. Importantly, we are not attempting to actually predict future mortality rates but rather using our forecasts to illustrate the effect that uncertainties could have on such predictions.

To accomplish these tasks we used data from long-term forest plots arrayed along a steep elevational gradient in California’s Sierra Nevada mountain range. Earlier analyses of these data revealed that tree mortality rates had increased significantly over the last two decades and that, for all elevations combined, these increases were correlated with temperature-driven increases in climatic water deficit. These previous analyses also ruled out competition, fire suppression, air pollution, self-thinning, and aging as confounding factors [9], [24]. This unique longitudinal data set is ideal for our current purposes because (i) samples are large and of fine temporal resolution, with the fates of >20,000 individual trees tracked annually for up to 24 years, and (ii) the forests were sampled along a 1900 m elevational gradient, ranging from water-limited forests near lower treeline to energy-limited forests at upper treeline.

## Materials and Methods

### Study Sites and Tree Mortality Rates

Twenty-one permanent study plots ranging in size from 0.9 to 2.5 ha were established between 1982 and 1996 in old-growth stands within the coniferous forests of Sequoia and Yosemite national parks, Sierra Nevada, California (Table S1). These forests have mixed age and size structure, with trees ranging from recent recruitment to mature canopy trees (Fig. S1). The plots have not experienced stand-replacing disturbances in at least two centuries, and probably much longer (as estimated by counting rings on increment cores or nearby stumps, or by historical records and the sizes of the largest trees), and therefore the forests contain cohorts of all ages and all sizes (Fig. S1). A few other plots in our network were excluded due to recent disturbances (fire or avalanche). The plots are arranged along a steep elevational gradient (∼1900 m) from near lower to upper treeline and encompass several different forest types, including ponderosa pine-mixed conifer, white fir-mixed conifer, Jeffrey pine, red fir, and subalpine forests [25]. The sites have never been logged. Frequent, low severity fires characterized many of the forest types prior to Euro-American settlement, but the areas containing the study plots have not burned since the late 1800s [26]. The climate is montane mediterranean, with hot, dry summers and cool, wet winters in which ∼25–95% of annual precipitation (which averages 1100 to 1400 mm) falls as snow, depending on elevation [27]. Mean annual temperature declines sharply with elevation (∼5.2°C for every 1 km increase in elevation), ranging from roughly 11°C at the lowest plots to 1°C at the highest. Soils are relatively young (mostly inceptisols), derived from granitic parent material.

Within each plot all trees ≥1.37 m in height were tagged, mapped, measured for diameter, and identified to species. We censused all plots annually for tree mortality, and at intervals of ∼5 years we remeasured diameter at breast height (dbh, 1.37 m above ground level) of living trees and recorded new recruitment. Data selection and mortality rate calculations followed van Mantgem and Stephenson [9] with the exception that mortality rates were calculated through 2006, yielding a final data set of 21,024 trees for analysis. Mortality rates were calculated only for the 86% of tree mortalities that were not the result of mechanical factors (uprooting, breaking, or being crushed), since mechanical mortalities are likely to be only indirectly associated with the climatic factors considered here [9]. This gave a total of 3788 mortalities for the period of record. Summary data are provided in Table S2.

### Ethics Statement

Research was performed in Sequoia National Park and Yosemite National Park. All necessary permits were obtained from the National Park Service for this study, which complied with all relevant regulations.

### Defining Water- vs. Energy-Limited Forests

Tague *et al.* [28] used a coupled ecohydrologic model to forecast the effects of temperature changes on net primary productivity (NPP) in the central Sierra Nevada, finding that increasing temperature resulted in declining NPP below about 2200 m elevation but increasing NPP above about 2600 m elevation, suggesting that the transition from water limitation to energy limitation occurs between about 2200 m and 2600 m elevation. Similarly, the response of remotely-sensed forest greening in the Sierra Nevada to interannual variation in snowpack water content suggests that the transition from water to energy limitation occurs between about 2100 m to 2600 m elevation [29]; these results were independently corroborated by *in situ* flux tower measurements at 2015 and 2700 m elevation [29]. Thus, although the transition from predominantly water-limited (low elevation) to predominantly energy-limited (high elevation) forests is likely to be gradual, different lines of evidence are consistent in suggesting that the midpoint of the transition falls near 2400 m elevation. We chose to define the transition at 2450 m, as this elevation allowed us to cleanly segregate the plots we used in model-building by forest types: ponderosa pine-mixed conifer, white fir-mixed conifer, and Jeffrey pine forests (≤2450 m, predominantly water-limited), and red fir and subalpine forests (>2450 m, predominantly energy-limited).

### Climatic Data

We obtained historical values of monthly-averaged precipitation and air temperature in a gridded map format at a 4-km spatial scale from the empirically-based Parameter-Elevation Regressions on Independent Slopes Model (PRISM) [30]. Spatial downscaling was performed on the coarse resolution grids (4 km) to produce fine resolution grids (270 m) using a model developed by Nalder and Wein [31] modified with a “nugget effect” specified as the length of the coarse resolution grid [32].

This technique combines a spatial Gradient and Inverse Distance Squared (GIDS) weighting to monthly point data using multiple regressions calculated for every grid cell for every month. Using the 4-km resolution digital elevation model in PRISM, parameter weighting is based on the location and elevation of the new fine resolution grid (270 m for this study) relative to existing coarse resolution grid cells [32]. The monthly maps of precipitation and minimum and maximum air temperature are used as input to a monthly water balance model (the Basin Characterization Model) to calculate potential and actual evapotranspiration and climatic water deficit for the Sierra Nevada at a grid spacing of 270 m.

### Climatic Water Deficit from the Basin Characterization Model

Climatic water deficit is defined as *PET-AET*, where *PET* is potential evapotranspiration and *AET* is actual evapotranspiration. Deficit explicitly considers the seasonal interactions of temperature and precipitation, as well as the timing of snowmelt and the soil water holding capacity. Deficit is therefore a more biologically relevant measure of water stress than other commonly used indices [4],[5].

To calculate deficit we used the Basin Characterization Model (BCM): a physically-based model that calculates water balance fractions based on data inputs for topography, soil composition and depth, underlying bedrock geology, and spatially-distributed values (measured or estimated) of air temperature and precipitation [33], [34]. The BCM calculates monthly recharge and runoff using a deterministic water-balance approach based on the distribution of precipitation and the estimation of potential evapotranspiration [33], [34] using the Priestley-Taylor equation [35]. The BCM relies on rigorous hourly energy balance calculation (used for the Priestley-Taylor equation) using topographic shading and applies available spatial maps of elevation, bedrock permeability estimated from geology, soil water storage from STATSGO and SSURGO soil databases [36], vegetation density, and PRISM maps of precipitation and minimum and maximum air temperature.

The BCM was calibrated regionally to measured potential evapotranspiration data and MODIS snow cover data [37]. Locally, the model was also calibrated to measured unimpaired streamflow data [34]. The determination of whether excess water becomes recharge or runoff is governed in part by the underlying bedrock permeability. The higher the bedrock permeability, the higher the recharge and the lower the runoff generated for a given grid cell. In small gaged basins that generate unimpaired flows, the bedrock permeability can be adjusted to calculate a total basin discharge that matches the measured basin discharge.

### Tree Mortality Rate Model Development

We developed statistical models relating changes in tree mortality rate to changes in temperature and climatic water deficit. Changes were calculated relative to reference period averages. For mortality rate, the reference period for a given plot was the period of record for that plot (plot establishment through 2006). For climate, the reference period was the water years (October 1 to September 30) 1982 through 2006 – the full range of years of record for all plots combined. (Note that mortality rates could be calculated only for the period of record of a given plot, whereas climatic values could be calculated for longer periods).

To account for systematic variation in tree mortality rates with elevation in the Sierra Nevada [38] and to facilitate interpretation of results, we modeled relative rather than absolute changes in mortality rates, with change defined against the reference period rate. Specifically, relative change in mortality rate for each plot was calculated as
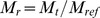
(1)where *M_r_* is the relative change in mortality rate, *M_t_* is mortality rate for the year *t,* and *M_ref_* is the reference mortality rate (i.e., the average mortality rate for the period of record of the given plot). As a check, we also modeled absolute changes in mortality rates and found no qualitative changes to our results.

The effects of temperature on biological processes are generally expressed in terms of absolute rather than relative changes in temperature (e.g., [11], [39]) so we used absolute change in temperature as an independent variable:
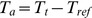
(2)where *T_a_* is the absolute change in average annual temperature, *T_t_* is the annual average temperature in year *t,* and *T_ref_* is the reference average annual temperature. As a check we also modeled using relative changes in temperature [in K] and found no qualitative changes to our results.

We could identify no *a priori* biological basis for choosing between relative versus absolute changes in deficit for use as independent variables (see the Introduction), so we used both to test whether one or the other were better predictors of change in mortality rate. These were calculated as
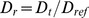
(3)

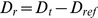
(4)where *D_r_* and *D_a_* are, respectively, the relative and absolute changes in climatic water deficit, *D_t_* is the deficit for year *t,* and *D_ref_* is the average deficit in the given plot for the reference period.

To allow for lagged and cumulative effects, we also calculated running averages of *D_r_*, *D_a_*, and *T_a_* that incorporated the current year and the prior one to four years (i.e., average of current year and prior one to four years). Running averages were also calculated that excluded the current year.

The relationships between relative change in mortality and climatic variables were modeled as either linear or exponential functions:
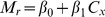
(5)

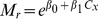
(6)where C*_x_* is the given climatic variable (*D_a_*, *D_r_*, or *T_a_*, including running averages of those variables), and β_0_ and β_1_are estimated parameters. Additionally, logistic functions were tried but discarded because uncertainty in the upper asymptote resulted in unstable parameter estimations (i.e., there was not enough information in the data to reliably estimate a maximum increase in mortality rate). Models were parameterized separately for all forests combined, water-limited forests (≤2450 m in elevation), and energy-limited forests (>2450 m elevation).

To account for site effects, we initially used mixed-effect models with site as a random effect. In the vast majority of cases, however, the estimated site effect was negligible and gave parameter values for the fixed effects that were statistically indistinguishable from those estimated using a fixed effects model. This was likely due to our standardization of climate and mortality values to a reference period. The mixed effects models also gave higher Akaike’s Information Criterion (AIC, see below) values, indicating less support. Even in the very few cases where the mixed effects term was non-trivial, parameter estimates for the fixed effects were statistically indistinguishable from their fixed effects model counterparts and AIC model rankings were the same. In addition, maximum likelihood estimations for the mixed effects parameters tended to be highly sensitive to starting values. Therefore, given that mixed effects terms did not appear to offer any improvement to or have any meaningful effect on the analysis, we proceeded with standard fixed effects models.

Models were fit using a maximum likelihood approach. Relative changes in mortality rates were treated as counts with the previous year’s total count of live trees used as an offset. For example, for linear models the count was given by:
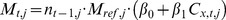
(7)Where *M_t,j_* is the count of mortalities at year *t* in plot *j*; *n_t-_*_1,*j*_ is the count of live trees at year *t-*1 in plot *j*; *M_ref,j_* is the reference mortality rate in plot *j*; and *C_x,t,j_* is the given climatic variable at time *t* in plot *j*. Parameters for these equations were then fit by maximizing a negative binomial log likelihood function using SAS 9.1 (SAS Institute. 2004. SAS OnlineDoc® 9.1.3. Cary, NC: SAS Institute Inc).

Models were compared with an information theoretic approach using AIC [40] to determine which type of model (linear or exponential) and which climatic parameter (*D_a_, D_r_,* and *T_a_*) best predicted mortality rate for each elevational class. As a guide, based upon Burnham and Anderson’s [40] rules of thumb, ΔAIC values less than 2 or an evidence ratio less than 2.7 would be considered very little evidence that one model is better than another while a ΔAIC greater than 4 or an evidence ratio greater than 7.4 would be considered relatively strong evidence for one model being better than another. The highest ranked models for each elevational class were then chosen and used for forecasting change in future tree mortality rates under different climate scenarios, described below.

### Mortality Forecasting

Note that the forecasts we perform here are intended to illustrate potential pitfalls in mortality forecasting given uncertainty in model choice. They are not intended nor should be interpreted as an attempt to reliably predict future mortality rates. For this illustration, changes in tree mortality rates in the coniferous forests of the Sierra Nevada were forecast through year 2100. We chose to use Sierra Nevada conifer forests and climate forecasts derived from General Circulation Models (GCM) in order to provide a biologically-relevant and biologically-plausible context for our illustration, but we might just have easily chosen a hypothetical dataset with arbitrarily chosen values.

The extent of coniferous forests was determined from the California Gap Analysis Project’s primary Wildlife Habitat Relationship habitat types (WHR1) [41]. On a one km resolution grid, this resulted in the selection of 33,594 grid points within the Sierra Nevada ecoregion (as defined by Hickman [42]) encompassing an area of approximately 33,600 km^2^.

Grid points were classified by elevation as containing either predominantly water- or predominantly energy-limited forest (≤2450 m or >2450 m elevation, respectively). Of course, the elevational boundary between water- and energy-limited forests almost certainly varies with latitude, topography, rain shadow effects, and other factors. However, we opted for this simple classification by elevation because our primary goal was to gain broad, qualitative insights from model comparisons, not to predict future conditions on a particular piece of ground. As an additional check, we performed forecasts in which we classified grid points as containing either water- or energy-limited forest based on forest type rather than elevation, and these forecasts showed no qualitative differences from those based on elevation that we report here.

### Downscaling Future Climate Scenarios

GCM climatic forecasts are generally available for the continental US at 12 km spatial resolution. A set of these projections have been downscaled for California and its environs using the constructed analogs method of Hidalgo et al. [43], providing a basis for our further downscaling [32]. Our goal was to represent climate projections for California on the basis of global climate models that have proven capable of simulating recent historical climate, particularly the distribution of monthly temperatures and the strong seasonal cycle of precipitation that exists in the region [44]–[46]. In addition, models were selected to represent a range of model sensitivity to greenhouse gas forcing. On the basis of these criteria, two GCMs were selected, the Parallel Climate Model (PCM) developed by National Center for Atmospheric Research (NCAR), Department of Energy (DOE) (see [47], [48]) and the National Oceanic and Atmospheric Administration (NOAA) Geophysical Fluid Dynamics Laboratory CM2.1 model (GFDL) [49], [50]. The choice of greenhouse gas emissions scenarios included A2 (medium-high–essentially “business as usual”) and B1 (low-essentially a “mitigated emissions” scenario), was guided by considerations presented by Nakicenovic *et al.* [51]. Thus we developed a range of climatic forecasts based on four specific scenarios; two models each driven by two emissions scenarios: “GFDL A2”, “GFDL B1”, “PCM A2”, “PCM B1”.

These four scenarios were downscaled from the 12-km grid scale to the historical PRISM data scale of 4 km for the purpose of bias correction. To make the correction possible the GCM was run for a historical forcing function to establish a baseline for modeling to match current climate. The baseline period for this study was defined as the PCM and GFDL model runs for 1950–2000, representing current (pre-2000) atmospheric greenhouse gas conditions. This baseline period was then adjusted using the PRISM data from 1950–2000, for each month and for each grid cell. Our approach to bias correction is a simple scaling of the mean and standard deviation of the projections to match those of the PRISM data following Bouwer *et al.* [52] and described in detail in Flint and Flint [32]. Once the bias correction was complete, the 4 km projections were further downscaled to 270 m spatial resolution using the GIDS spatial interpolation approach for model application.

Changes in tree mortality rate were forecast for each year for each grid point under each combination of GCM and emissions scenario by inputting forecasted climate into our best mortality models. Climate and mortality forecasts were then summarized by decade for each grid point.

## Results

### Model Selection and Comparison

For all elevations combined and for low elevation forest plots (≤2450 m), the strongest predictors of 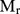
were running averages of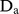,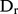, and *T_a_* that included the current year change in deficit or temperature plus either the prior two years or three years (the latter only for low elevation temperature models) (Table 1). Deficit models had substantially stronger support than temperature models. In contrast, the evidence was at best equivocal in distinguishing between absolute or relative deficit variables as the better predictors of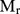. Thus, while both types of deficit variables appear to be better predictors of changes in mortality than temperature variables at all elevations combined and at low elevations, there was no strong support for one type of deficit variable over the other. In all cases, ΔAIC indicated roughly equal support for both linear and exponential models.

**Table 1:** Top Ranked Models and Parameters.

Predictor Variable	Model Form	ΔAIC	Evidence Ratio	β_0_		β_0_ S.E.		β_1_		β_1_ S.E.		α		α S.E.	
*All Elevations* (21 plots)															
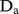 *(Absolute Change)*															
Current year plus two prior	Exponential	0.0	1.0	−0.0536			0.0321		0.0036		0.0005		0.1709		0.0246
Current year plus two prior	Linear	0.6	1.3	0.9726			0.0305		0.0034		0.0005		0.1711		0.0246
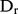 *(Relative Change)*															
Current year plus two prior	Exponential	2.9	4.3	−1.2159		0.1774		1.1643		0.1675		0.1752		0.0249	
Current year plus two prior	Linear	3.8	6.7	−0.1257		0.1500		1.0989		0.1537		0.1756		0.0250	
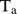 *(Absolute Change)*															
Current year plus two prior	Exponential	15.7	2565.7	−0.0823	0.0350				0.5515		0.0939		0.1870		0.0259
Current year plus two prior	Linear	14.3	1274.1	0.9388	0.0308				0.5325		0.0829		0.1856		0.0258
*Low Elevation (Water-limited)* (13 plots)															
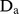 *(Absolute Change)*															
Current year plus two prior	Exponential	0.0	1.0	−0.0507		0.0356		0.0036		0.0006		0.1697		0.0263	
Current year plus two prior	Linear	0.2	1.1	0.9748		0.0339		0.0034		0.0005		0.1697		0.0264	
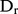 *(Relative Change)*															
Current year plus two prior	Exponential	2.3	3.2	−1.3272		0.2170		1.2783		0.2066		0.1728		0.02659	
Current year plus two prior	Linear	3.0	4.5	−0.2212		0.1802		1.1965		0.1848		0.1735		0.02670	
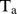 *(Absolute Change)*															
Current year plus three prior	Exponential	9.3	104.6	−0.0752		0.03843		0.6851		0.1261		0.1881		0.0279	
Current year plus three prior	Linear	7.0	33.1	0.9465		0.03431		0.6695		0.1033		0.1856		0.0276	
*High Elevation (Energy-limited)* (8 plots)															
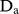 *(Absolute Change)*															
Current year plus two prior	Exponential	0.5	1.3	−0.0661		0.0751		0.0037		0.0011		0.1783		0.0686	
Current year plus two prior	Linear	0.8	1.5	0.9638		0.0703		0.0035		0.0011		0.1799		0.0689	
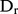 *(Relative Change)*															
Current year plus two prior	Exponential	0.0	1.0	−1.0287		0.3133		0.9609		0.2863		0.1777		0.0684	
Current year plus two prior	Linear	0.4	1.2	0.0648		0.2644		0.8986		0.2711		0.1793		0.0687	
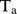 *(Absolute Change)*															
Current year plus four prior	Exponential	3.6	6.0	−0.1481		0.0900		0.7864		0.2846		0.1829		0.06742	
Current year plus four prior	Linear	3.8	6.7	0.8764		0.0734		0.7534		0.2740		0.1822		0.06766	

Note: ΔAIC is the difference in AIC value between the top ranked model and the given model. Smaller values indicate better models. Evidence Ratio can be interpreted as how much stronger the evidence is for the top ranked model over the given model. Larger values indicate stronger evidence that the top ranked model is better than the given model. β_0_ and β_0_ S.E. are estimated intercept parameter and standard error for the model (see Eqns. 5–7). β_1_ and β_1_ S.E. are estimated parameter and standard error for the deficit variable for the model (see Eqns. 5–7). α is a parameter from the negative binomial distribution that quantifies over dispersion relative to a Poisson distribution, where the over dispersion factor is defined as (1+1/α). Therefore, smaller values of α represent larger over dispersion.

For high elevation forest plots (>2450 m), the strongest predictors of 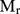 were running averages that included the current year plus the prior two or four years (Table 1). Deficit models were still top ranked, but ΔAIC values and evidence ratios indicate that they had only marginally more support than the best *T_a_* models. In all cases, ΔAIC indicated roughly equal support for both linear and exponential models.

Although temperature and deficit variables are correlated, the strength of that correlation (between *D_a_* and *T_a_* including the current plus two prior years) was indistinguishable between low and high elevations (r = 0.68 for low elevations, r = 0.64 for high elevations,). The increased relative predictive power of temperature at high elevation therefore cannot be explained by increased correlation between temperature and deficit.

In comparing the models developed for each set of data (all elevations, low elevations, and high elevations), the similarity in the parameter estimates for the deficit models, particularly the *D_a_* models, is notable, suggesting that the relationship between changes in mortality rate and changes in deficit does not vary substantially across a very marked elevational gradient.

### Forecasted Changes in Mortality Rates

Temperature increased for all combinations of GCMs and emissions scenarios, with average increases between the 2000s to the 2090s ranging from 1.2 to 4.2°C (Fig. S2). The GFDL model tended to forecast a general decrease in precipitation in the Sierra Nevada, while the PCM model showed no straightforward pattern (Fig. S2). Climatic water deficit increased in all cases, with the GFDL A2 combination showing the sharpest increase (Fig. S2).

All mortality models forecast increasing tree mortality rates throughout the coniferous forests of the Sierra Nevada, with spatially-averaged projected increases by the 2090s varying dramatically depending on the model used and position on the landscape (see below). In general, the GFDL model resulted in larger increases in mortality rates than the PCM model, and the A2 scenario resulted in larger increases than the B1 scenario.

**Figure 1:**
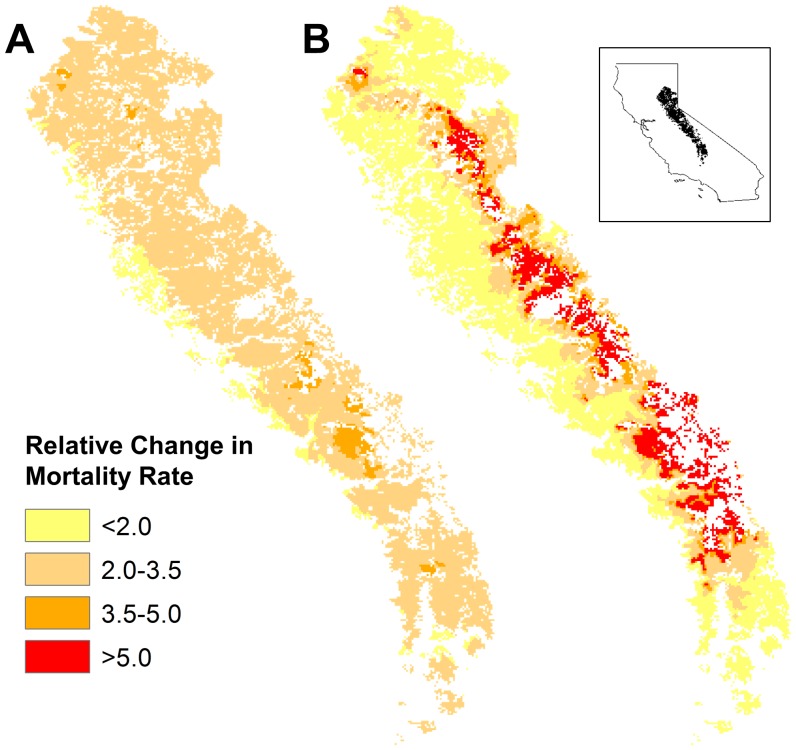
Projected Changes in Mortality Rate for Sierra Nevada Conifer Forests (Hypothetical). Mapped projections of average relative changes in mortality rate for the years 2090 to 2099 for coniferous forests of California’s Sierra Nevada, using exponential and the GFDL A2 emissions model. Elevations generally increase from left to right. (A) Changes in mortality when absolute changes in deficit (*D_a_*) are used as a predictor. (B) Changes in mortality when relative changes in deficit (*D_r_*) are used as a predictor. Surfaces were interpolated from 33,594 grid points using Ordinary Kriging. Other emissions scenarios gave qualitatively similar results (Figs S3 to S5).

**Figure 2:**
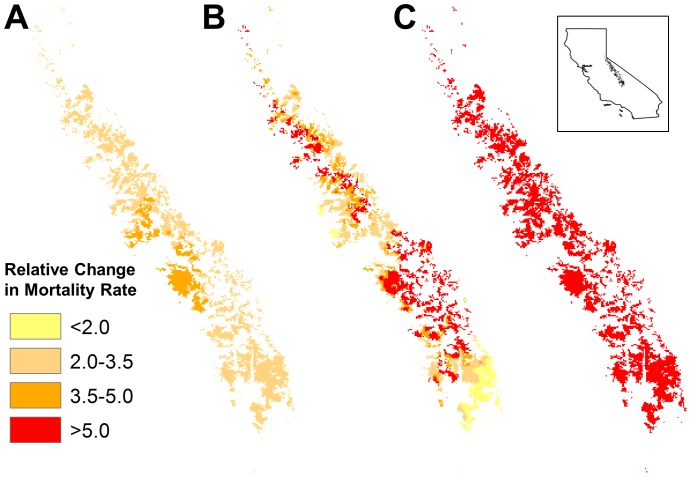
Projected Changes in Mortality Rate for predominantly Energy-Limited Sierra Nevada Conifer Forests (Hypothetical). Mapped projections of average relative changes in mortality rate for the years 2090 to 2099 for energy-limited coniferous forests (≥2450 m) of California’s Sierra Nevada, using exponential models and the GFDL A2 emissions model. Elevations generally increase from left to right. (A) Changes in mortality when absolute changes in deficit (*D_a_*) are used as a predictor. (B) Changes in mortality when relative changes in deficit (*D_r_*) are used as a predictor. (C) Changes in mortality when absolute changes in temperature (T*_a_*) are used as a predictor. Other emissions scenarios gave qualitatively similar results (Figs. S6 to S8).

**Figure 3:**
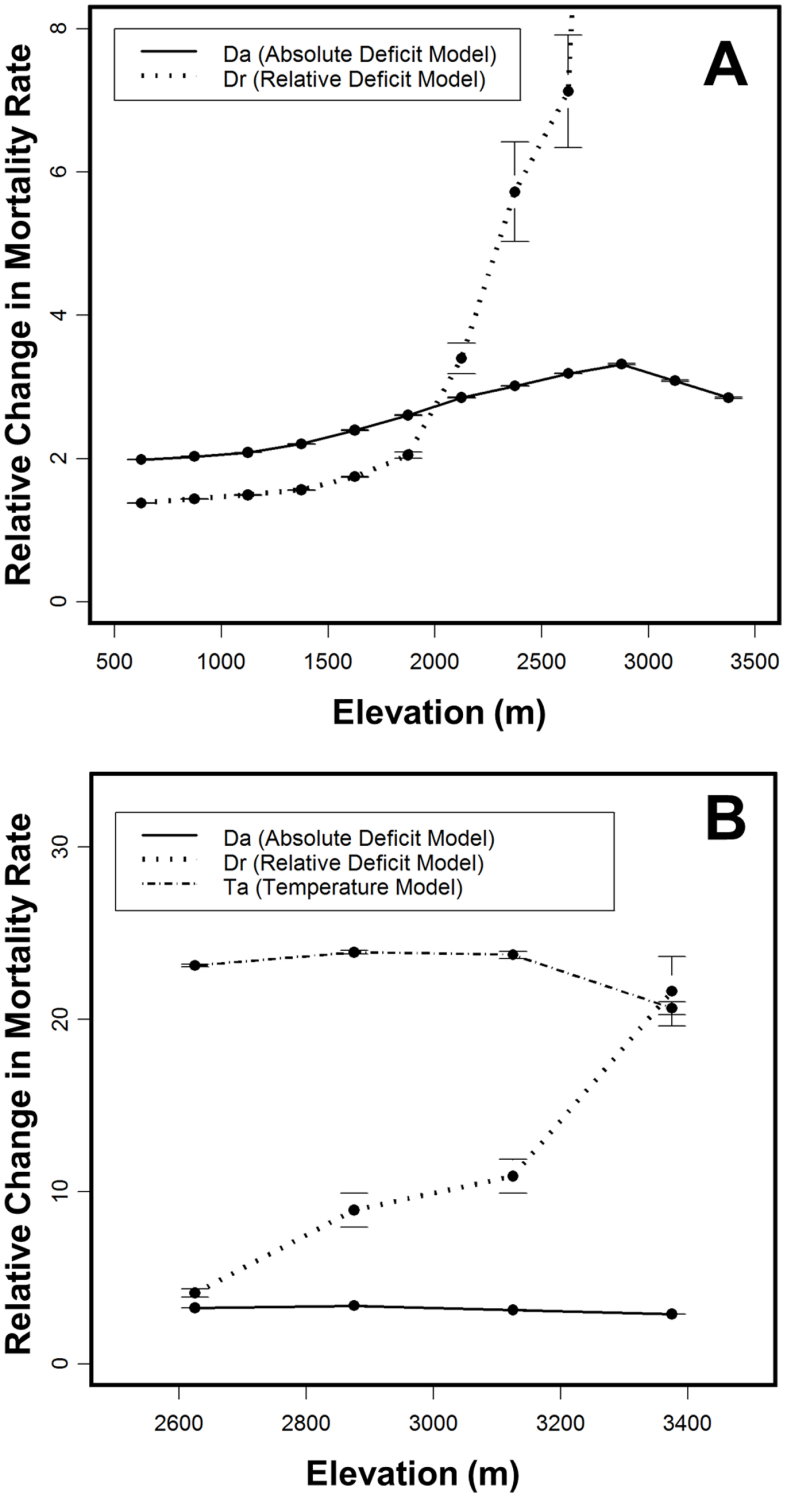
Projected Changes in Mortality Rate by Elevation (Hypothetical). Projected relative changes in tree mortality rate by elevation in the 2090s for the GFDL A2 climatic scenario using (A) *D_a_* and *D_r_* exponential models for all forests and (B) the *D_a_, D_r_,* and *T_a_* exponential model for energy-limited forests. Rates are averaged in 250 m classes using the 33,594 Sierra Nevada coniferous forest grid points. Error bars show standard error. Note that the scales on A and B are different. A small number of points with small baseline deficit values (<1% of all points for A and <2% for B) were excluded because the *D_r_* models predicted exceedingly large changes in mortality rate. Projections using the PCM model and other emission scenarios as well as using linear mortality models gave qualitatively similar results.

The spatial pattern of mortality rate increases differed markedly among forecasts from the *D_a_, D_r_,* and *T_a_* models (Fig. 1, Fig. 2, Fig. 3). In particular, the models make dramatically different forecasts about how mortality rate will change along elevational gradients. *D_a_* models forecast gradually increasing mortality rates with elevation (Fig. 1A, Fig. 2A, Fig. 3), reflecting modestly increasing changes in absolute deficit with elevation. In contrast, *D_r_* models forecast smaller increases in mortality rates at lower elevations but very sharply increasing rates for forests above about 2200 m (Fig. 1B, Fig. 2B, Fig. 3):a consequence of the fact that baseline deficits at higher elevations tend to be small and that relatively modest changes in absolute deficit can result in very large changes in relative deficit. Finally, in the elevational zone above 2450 m, where temperature was a relatively good predictor of changes in mortality rate, *T_a_* models forecast large and relatively uniform increases in mortality rate at all elevations within the zone, except at the highest elevations where the magnitude of increase drops somewhat (Fig. 2C, Fig. 3B). This pattern tracks the forecasted changes in temperature at different elevations.

## Discussion

### Deficit versus Temperature

To our knowledge, we provide the first explicit correlative test of the effects of climate on tree mortality in water- versus energy-limited forests. As expected, changes in mortality rate in water-limited forests (low elevations) were unambiguously best modeled by deficit. In energy limited forests (high elevation) temperature also became a relevant predictor, with deficit models providing only marginally better fits.

Two factors might account for the fact that temperature variables, while stronger predictors at higher elevations, were not unambiguously the best predictors at those elevations: (i) energy and water limitation are probably more properly described as a continuum rather than as a dichotomy, and (ii) the proposed mechanisms driving increasing tree mortality rates (increased drought stress and favorability to enemies) are not mutually exclusive. Therefore, it is not surprising that deficit might still be an important driver of mortality rates at higher elevations, even if it becomes less clearly the best predictor.

On the first point, we have assumed a simple dichotomy between water- and energy-limited forests. In reality, water and energy limitation likely represent the extremes of graded variations in both space and time, with many forests being both water and energy limited (e.g., [53], [54], [55]). Additionally, with climatic change, as temperatures increase and winter snowpacks decrease through time, we expect that some forests will switch from being primarily energy-limited to primarily water-limited.

We also cannot eliminate the possibility that the observed difference in climatic correlates of tree mortality between our water- and energy-limited forests is a simple consequence of differences in the forests’ species composition. However, we believe this is unlikely. Importantly, both sets of forests are heavily dominated by the same two tree genera, *Abies* and *Pinus*, and are attacked by a similar (and sometimes identical) suite of pathogens and herbivores, suggesting that environmental effects rather than species effects provide the most parsimonious explanation of our results (e.g., [7], [8], [11], [12], [56]).

Regardless, our finding that temperature becomes a relatively stronger predictor of mortality rate in energy-limited forests agrees with recent studies examining climatic controls on tree growth, which have found that climatic controls of both individual tree growth [13] and overall forest productivity [57] vary depending on the water or energy limitation of a given forest. Littell *et al.* [13] for example found evidence that deficit drove tree growth patterns for most of the Douglas-fir forests in their study (i.e., growth was usually water-limited) but that at higher elevation sites growth appeared to be temperature- (energy-) limited.

Our findings have implications for our understanding of how forest structure, dynamics, and carbon storage will change with a changing climate. Forests play a substantial role in the global carbon cycle [58], and changes in tree mortality can have dramatic effects on forest carbon dynamics (e.g., [1]). To date, however, most forest simulation models have relied on general mortality algorithms that do not incorporate explicit mechanisms and would therefore not be expected to adequately reflect changes in mortality processes across a gradient of water and energy limitation [59]–[61].

One encouraging result was the strong similarity in parameter estimations for deficit models in both low and high elevation forests. While it is likely that additional mechanisms will need to be considered across such gradients, relationships between drought and mortality may not vary substantially across fairly broad scales.

### Absolute versus Relative Deficit

Our inability to clearly distinguish among models using relative versus absolute changes in deficit as predictors of mortality also has critical implications for forecasting future changes in mortality rates. The two types of models lead to substantially different predictions of changes in mortality rates across the landscape (Figs. 1,2). If future studies yield similar results across latitudinal as well as elevational gradients, we could be confronted with drastically different conclusions about which forests are most at risk from a warming climate at subcontinental scales.

We currently have no strong *a priori* reasons for selecting one deficit model over another. Although critical groundwork has been laid for mechanistic models of drought-induced tree mortality [62], current models cannot yet tell us whether mortality is likely to be best predicted by absolute or relative changes in deficit. Empirical evidence is also equivocal. While some studies in areas of chronic water stress have found increased mortality during droughts (potentially providing opportunities to study the effects of increasing deficit [19], [63]–[65]), we do not yet have the data needed to assess whether trees in those regions were responding in a manner more consistent with absolute or relative changes in deficit. Additionally, it is not clear whether episodes of severe, short-term drought are directly comparable to the more gradual long-term increases in water stress that we are examining here. Distinguishing between the two models will likely require more definitive physiological models, larger empirical datasets that incorporate a wider range of deficits along gradients of water stress, and manipulative experiments that directly test the effect of changes in deficit.

### Additional Uncertainties

Given the uncertainties noted above, we emphasize that our forecasts of future tree mortality rates in the Sierra Nevada should not be taken as quantitative predictions but rather as an illustration of the dangers of attempting to predict tree mortality rates without a solid understanding of underlying mechanisms. As noted above, we chose to use the Sierra Nevada landscape and downscaled GCM projections in order to provide a biologically-plausible and biologically-relevant context for our illustration, but we might just as easily have used a completely hypothetical climate projection and a hypothetical forest.

Additional uncertainties should be acknowledged with regard to our forecasts. Of necessity, our models were developed from a range of deficit and temperature changes that is far smaller than the range projected for the future. It is therefore unlikely that we have captured the true shape of the relationship between deficit or temperature and mortality, as is indicated by our inability to distinguish between exponential and linear models. In addition, as noted above, mechanisms may change at a given location as temperatures increase. Perhaps most importantly, our models were parameterized on background (non-catastrophic) tree mortality rates and do not capture potential insect or pathogen outbreaks, such as those seen in recent years in many parts of western North America [12]. Our assumption of a simple monotonic relationship between climate and tree mortality is undoubtedly overly simplistic (see [10], [11]), and does not account for sudden threshold transitions that can result in extensive forest die-back. However, our forecasts serve their intended role of qualitatively illustrating how model choices can profoundly affect our ability to understand and predict future changes.

### Conclusions

Understanding the spatial relationships between climate and tree mortality is becoming increasingly important as climate continues to change. For example, van Mantgem *et al.* [24] found a widespread increase in mortality rates in old growth forests across the western USA and showed that regional warming is likely an important contributor to that increase. We have shown here that the mechanisms relating climate to tree mortality appear to vary along an elevational gradient, suggesting that a similar pattern may hold across a broader geographic scale, with the underlying mechanisms that drive changes in mortality perhaps varying between drier (water-limited) regions and wetter (energy-limited) regions. We have also demonstrated that identifying the precise nature of the relationship between climate variables and tree mortality can be critically important for making accurate predictions of forest change. At present, our ability to correctly identify such relationships is tenuous. A more thorough grasp of mortality mechanisms will be critical as we struggle to forecast and manage forest change in the decades to come.

## Supporting Information

Figure S1 Tree Size Distribution for Each Plot. Shows the size distribution of trees in each of the A) water-limited and B) energy-limited plots. The y-axis is given in a log base 10 scale.
(TIF)

Figure S2 Forecasted change in Sierra Nevada climate through time from different model scenarios. Climate data is averaged by decade for all 33,594 gridpoints. Gap between standard error bars for each point was too small to distinguish so they have not been included. A) temperature forecasts; B) precipitation forecasts; C) climatic water deficit forecasts.
(TIF)

Figure S3 Projected Changes in Mortality Rate for Sierra Nevada Conifer Forests (GFDL B1, Hypothetical). Mapped projections of average relative changes in mortality rate for the years 2090 to 2099 for coniferous forests of California’s Sierra Nevada, using exponential and the GFDL B1 emissions model. Elevations generally increase from left to right. (A) Changes in mortality when absolute changes in deficit (*D_a_*) are used as a predictor. (B) Changes in mortality when relative changes in deficit (*D_r_*) are used as a predictor. Surfaces were interpolated from 33,594 grid points using Ordinary Kriging.
(TIF)

Figure S4 Projected Changes in Mortality Rate for Sierra Nevada Conifer Forests (PCM A2, Hypothetical). Mapped projections of average relative changes in mortality rate for the years 2090 to 2099 for coniferous forests of California’s Sierra Nevada, using exponential and the PCM A2 emissions model. Elevations generally increase from left to right. (A) Changes in mortality when absolute changes in deficit (*D_a_*) are used as a predictor. (B) Changes in mortality when relative changes in deficit (*D_r_*) are used as a predictor. Surfaces were interpolated from 33,594 grid points using Ordinary Kriging.
(TIF)

Figure S5 Projected Changes in Mortality Rate for Sierra Nevada Conifer Forests (PCM B1, Hypothetical). Mapped projections of average relative changes in mortality rate for the years 2090 to 2099 for coniferous forests of California’s Sierra Nevada, using exponential and the PCM B1 emissions model. Elevations generally increase from left to right. (A) Changes in mortality when absolute changes in deficit (*D_a_*) are used as a predictor. (B) Changes in mortality when relative changes in deficit (*D_r_*) are used as a predictor. Surfaces were interpolated from 33,594 grid points using Ordinary Kriging.
(TIF)

Figure S6 Projected Changes in Mortality Rate for predominantly Energy-Limited Sierra Nevada Conifer Forests (GFDL B1, Hypothetical). Mapped projections of average relative changes in mortality rate for the years 2090 to 2099 for energy-limited coniferous forests (≥2450 m) of California’s Sierra Nevada, using exponential models and the GFDL B1 emissions model. Elevations generally increase from left to right. (A) Changes in mortality when absolute changes in deficit (*D_a_*) are used as a predictor. (B) Changes in mortality when relative changes in deficit (*D_r_*) are used as a predictor. (C) Changes in mortality when absolute changes in temperature (T*_a_*) are used as a predictor.
(TIF)

Figure S7 Projected Changes in Mortality Rate for predominantly Energy-Limited Sierra Nevada Conifer Forests (PCM A2, Hypothetical). Mapped projections of average relative changes in mortality rate for the years 2090 to 2099 for energy-limited coniferous forests (≥2450 m) of California’s Sierra Nevada, using exponential models and the PCM A2 emissions model. Elevations generally increase from left to right. (A) Changes in mortality when absolute changes in deficit (*D_a_*) are used as a predictor. (B) Changes in mortality when relative changes in deficit (*D_r_*) are used as a predictor. (C) Changes in mortality when absolute changes in temperature (T*_a_*) are used as a predictor.
(TIF)

Figure S8 Projected Changes in Mortality Rate for predominantly Energy-Limited Sierra Nevada Conifer Forests (PCM B1, Hypothetical). Mapped projections of average relative changes in mortality rate for the years 2090 to 2099 for energy-limited coniferous forests (≥2450 m) of California’s Sierra Nevada, using exponential models and the PCM B1 emissions model. Elevations generally increase from left to right. (A) Changes in mortality when absolute changes in deficit (*D_a_*) are used as a predictor. (B) Changes in mortality when relative changes in deficit (*D_r_*) are used as a predictor. (C) Changes in mortality when absolute changes in temperature (T*_a_*) are used as a predictor.
(TIF)

Table S1 Plot Details. Characteristics of the 21 forest plots used for model development.
(DOC)

Table S2 Plot Mortality Data. Counts of mortalities and live trees for each plot for each census year.
(DOC)
