# Supplementary figures and images for: Climatic Correlates of Tree Mortality in Water- and Energy-Limited Forests
Source: PLoS One. 2013 Jul 25;8(7):e69917. doi: 10.1371/journal.pone.0069917 (PMC3723662; doi:10.1371/journal.pone.0069917)

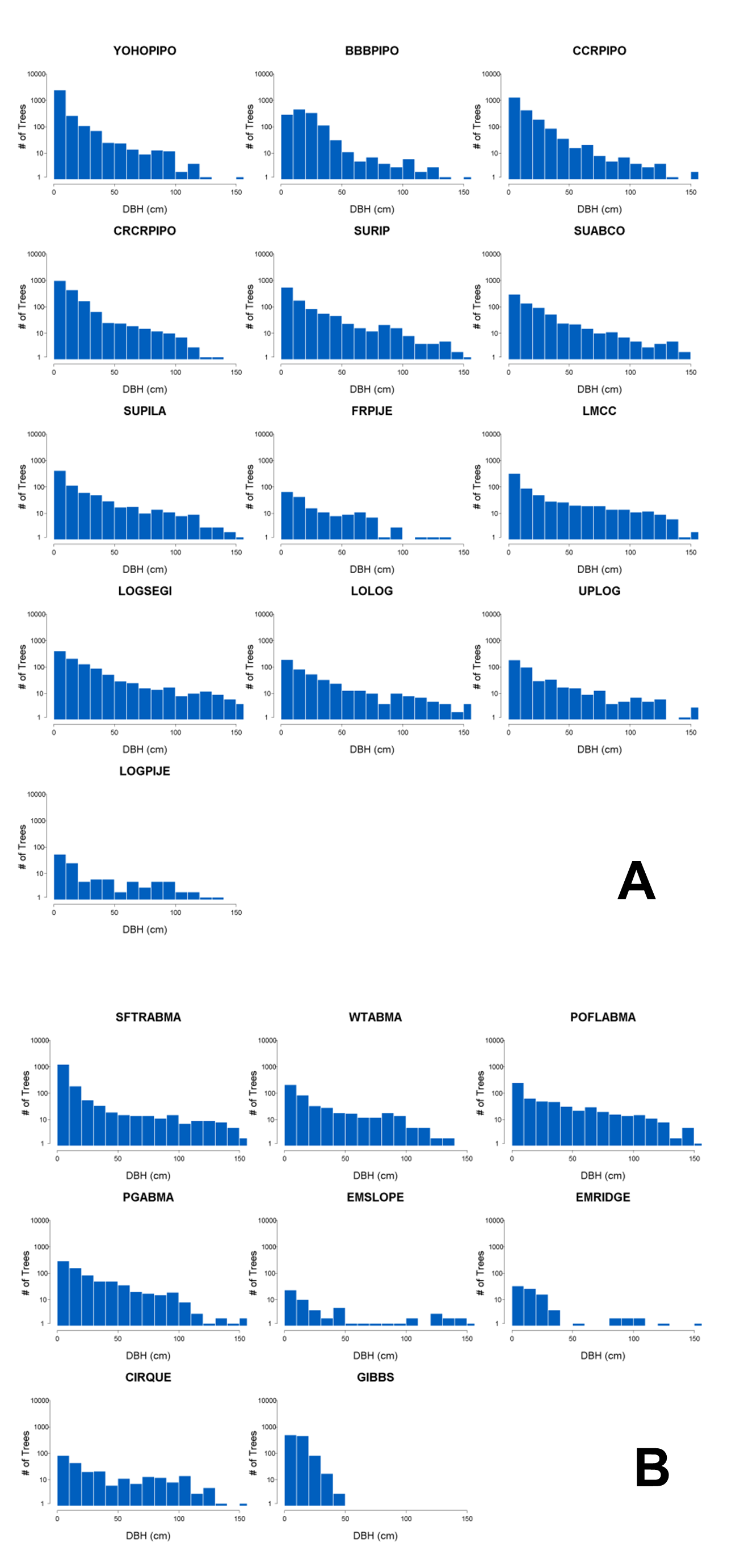

Supplement: Figure S1 — Tree Size Distribution for Each Plot. Shows the size distribution of trees in each of the A) water-limited and B) energy-limited plots. The y-axis is given in a log base 10 scale. (TIF) [file pone.0069917.s001.tif]

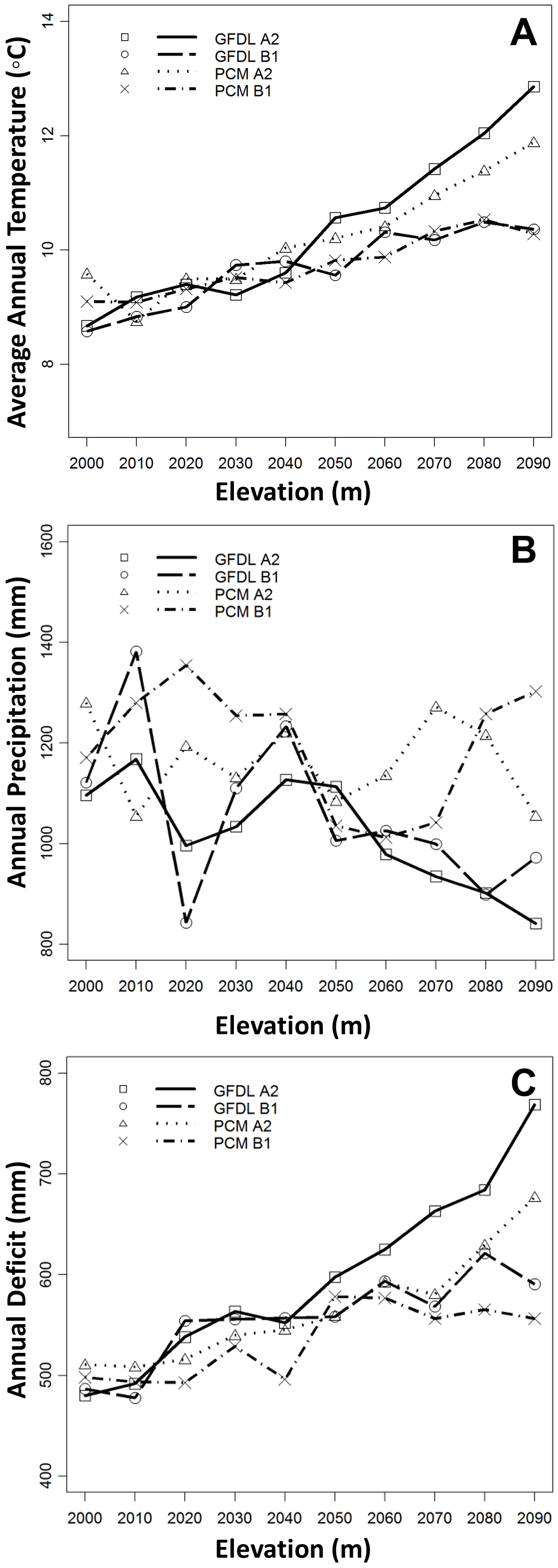

Supplement: Figure S2 — Forecasted change in Sierra Nevada climate through time from different model scenarios. Climate data is averaged by decade for all 33,594 gridpoints. Gap between standard error bars for each point was too small to distinguish so they have not been included. A) temperature forecasts; B) precipitation forecasts; C) climatic water deficit forecasts. (TIF) [file pone.0069917.s002.tif]

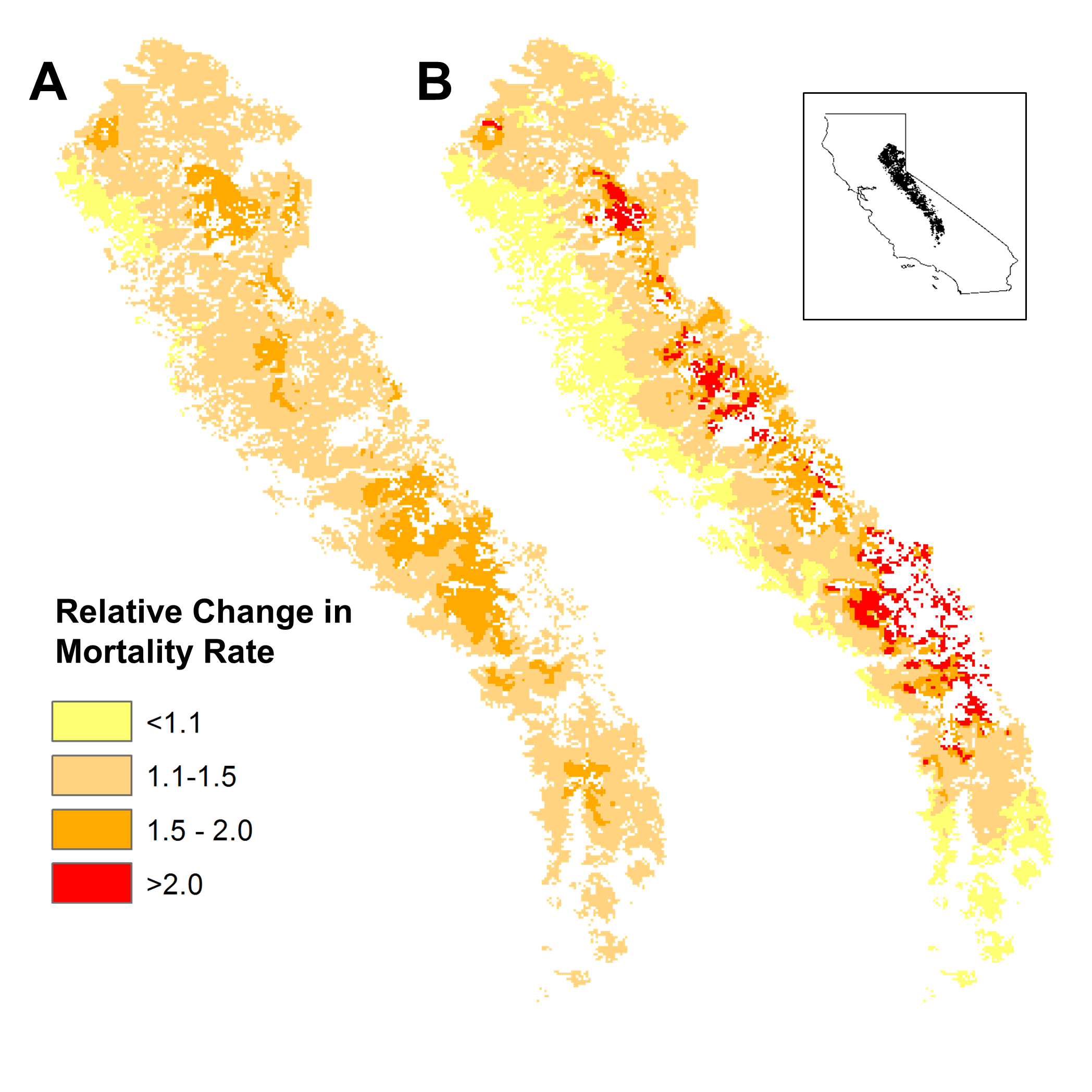

Supplement: Figure S3 — Projected Changes in Mortality Rate for Sierra Nevada Conifer Forests (GFDL B1, Hypothetical). Mapped projections of average relative changes in mortality rate for the years 2090 to 2099 for coniferous forests of California’s Sierra Nevada, using exponential and the GFDL B1 emissions model. Elevations generally increase from left to right. (A) Changes in mortality when absolute changes in deficit (Da) are used as a predictor. (B) Changes in mortality when relative changes in deficit (Dr) are used as a predictor. Surfaces were interpolated from 33,594 grid points using Ordinary Kriging. (TIF) [file pone.0069917.s003.tif]

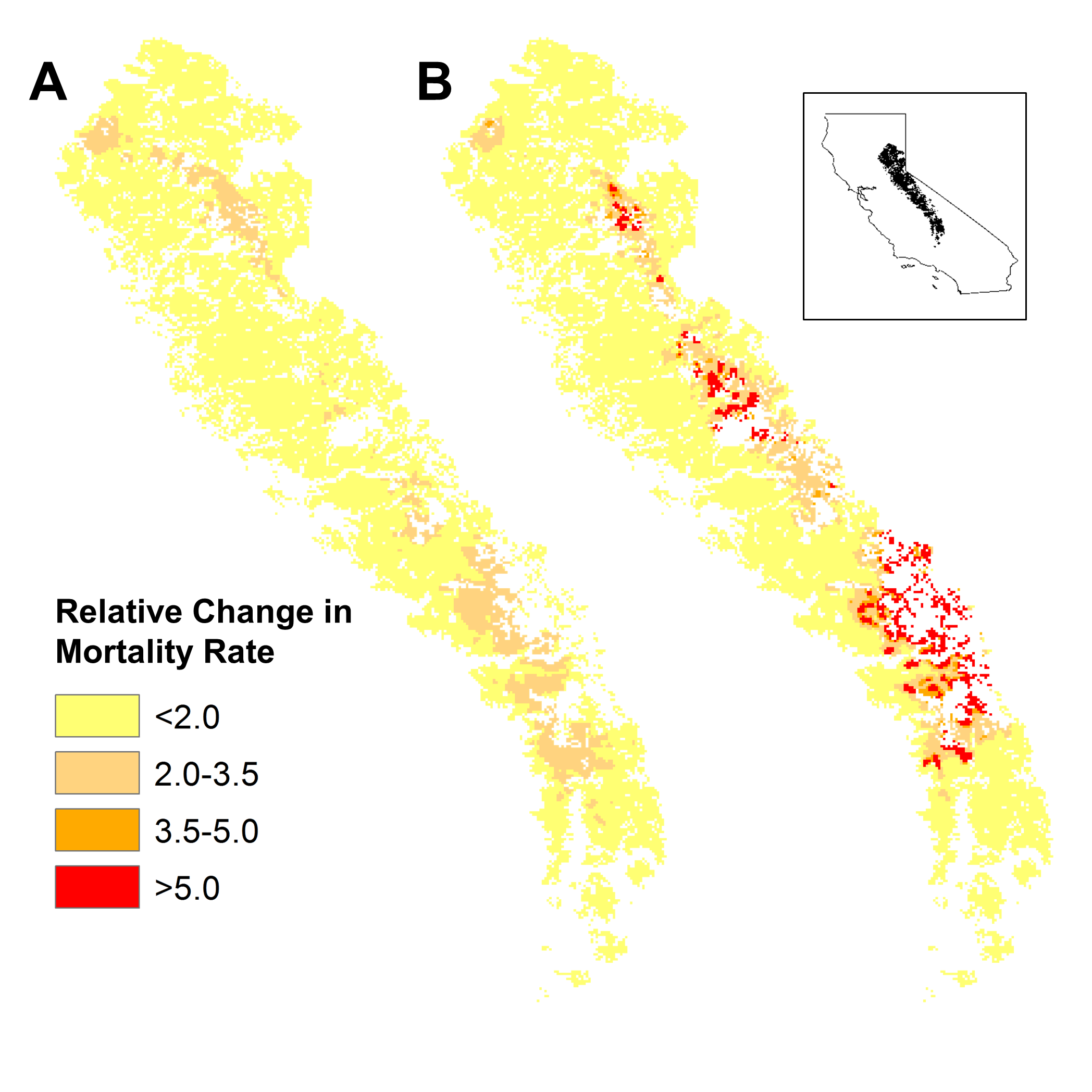

Supplement: Figure S4 — Projected Changes in Mortality Rate for Sierra Nevada Conifer Forests (PCM A2, Hypothetical). Mapped projections of average relative changes in mortality rate for the years 2090 to 2099 for coniferous forests of California’s Sierra Nevada, using exponential and the PCM A2 emissions model. Elevations generally increase from left to right. (A) Changes in mortality when absolute changes in deficit (Da) are used as a predictor. (B) Changes in mortality when relative changes in deficit (Dr) are used as a predictor. Surfaces were interpolated from 33,594 grid points using Ordinary Kriging. (TIF) [file pone.0069917.s004.tif]

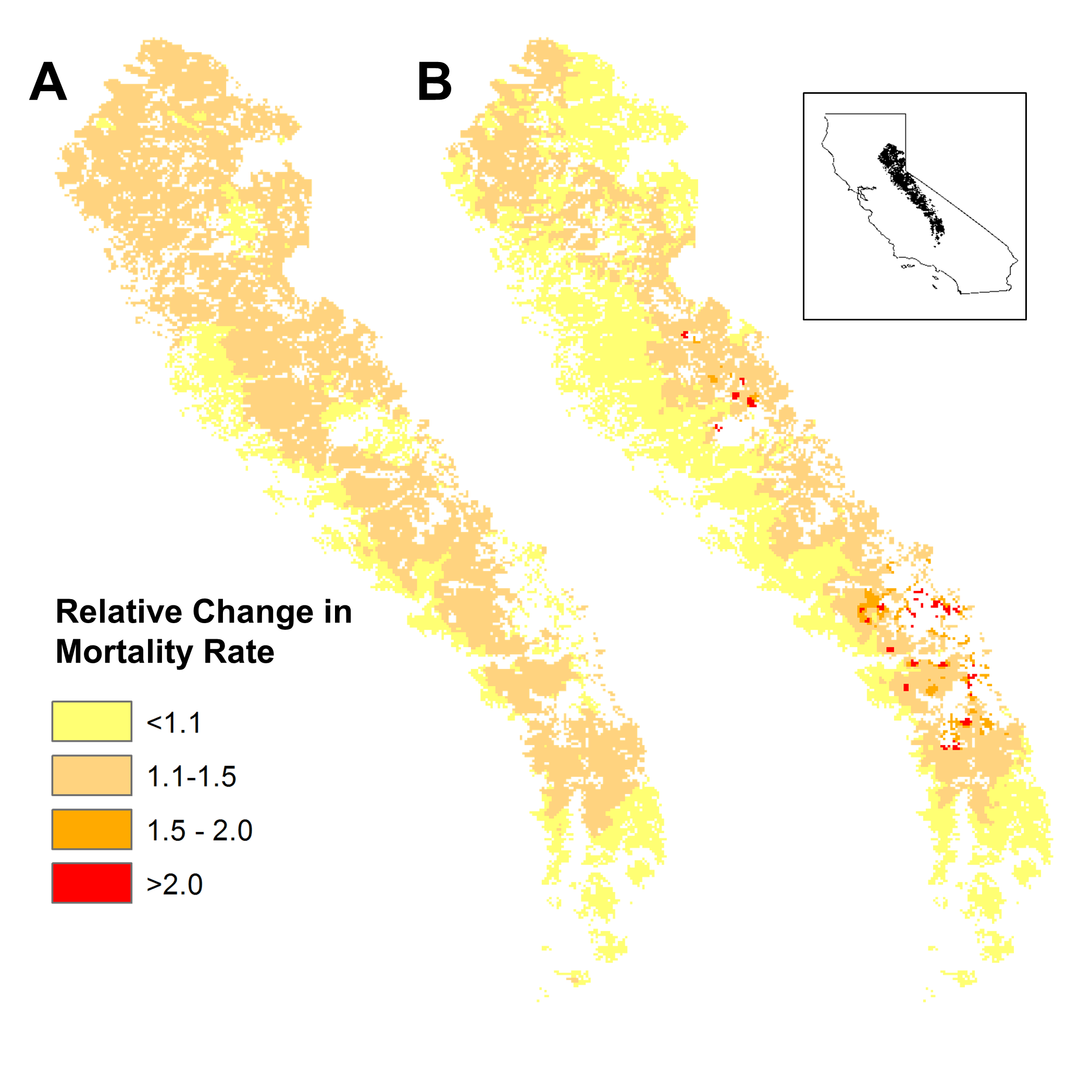

Supplement: Figure S5 — Projected Changes in Mortality Rate for Sierra Nevada Conifer Forests (PCM B1, Hypothetical). Mapped projections of average relative changes in mortality rate for the years 2090 to 2099 for coniferous forests of California’s Sierra Nevada, using exponential and the PCM B1 emissions model. Elevations generally increase from left to right. (A) Changes in mortality when absolute changes in deficit (Da) are used as a predictor. (B) Changes in mortality when relative changes in deficit (Dr) are used as a predictor. Surfaces were interpolated from 33,594 grid points using Ordinary Kriging. (TIF) [file pone.0069917.s005.tif]

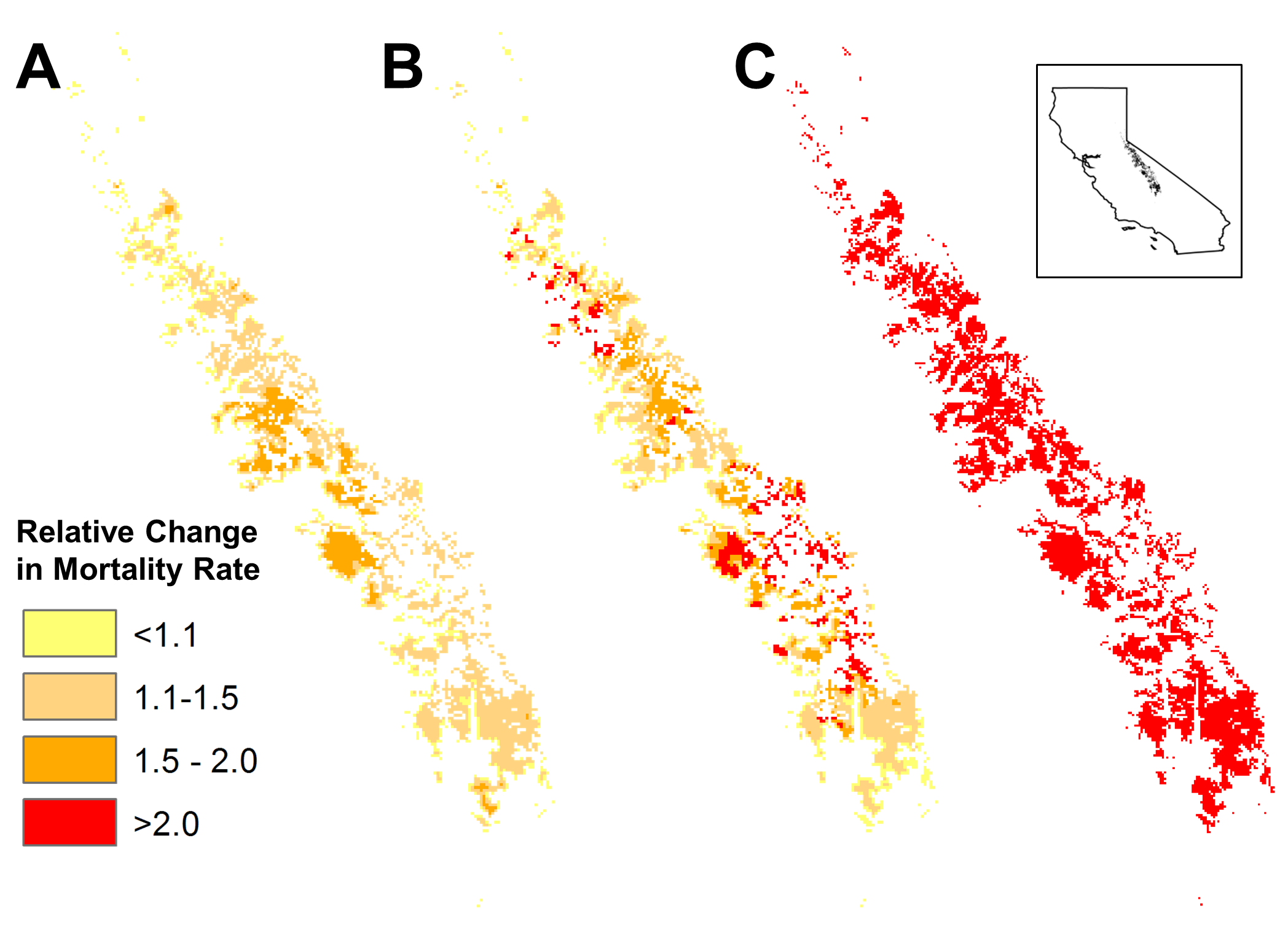

Supplement: Figure S6 — Projected Changes in Mortality Rate for predominantly Energy-Limited Sierra Nevada Conifer Forests (GFDL B1, Hypothetical). Mapped projections of average relative changes in mortality rate for the years 2090 to 2099 for energy-limited coniferous forests (≥2450 m) of California’s Sierra Nevada, using exponential models and the GFDL B1 emissions model. Elevations generally increase from left to right. (A) Changes in mortality when absolute changes in deficit (Da) are used as a predictor. (B) Changes in mortality when relative changes in deficit (Dr) are used as a predictor. (C) Changes in mortality when absolute changes in temperature (Ta) are used as a predictor. (TIF) [file pone.0069917.s006.tif]

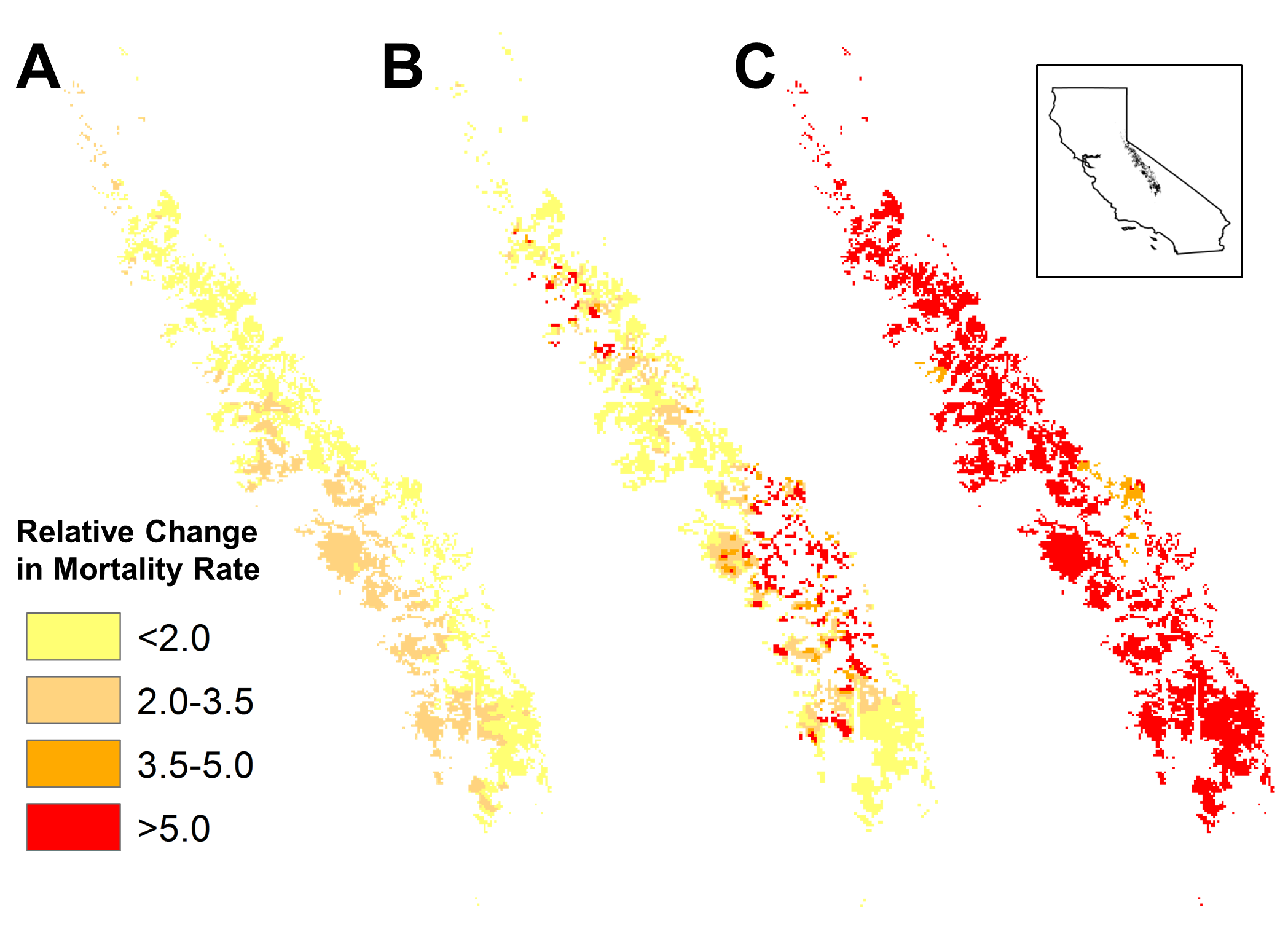

Supplement: Figure S7 — Projected Changes in Mortality Rate for predominantly Energy-Limited Sierra Nevada Conifer Forests (PCM A2, Hypothetical). Mapped projections of average relative changes in mortality rate for the years 2090 to 2099 for energy-limited coniferous forests (≥2450 m) of California’s Sierra Nevada, using exponential models and the PCM A2 emissions model. Elevations generally increase from left to right. (A) Changes in mortality when absolute changes in deficit (Da) are used as a predictor. (B) Changes in mortality when relative changes in deficit (Dr) are used as a predictor. (C) Changes in mortality when absolute changes in temperature (Ta) are used as a predictor. (TIF) [file pone.0069917.s007.tif]

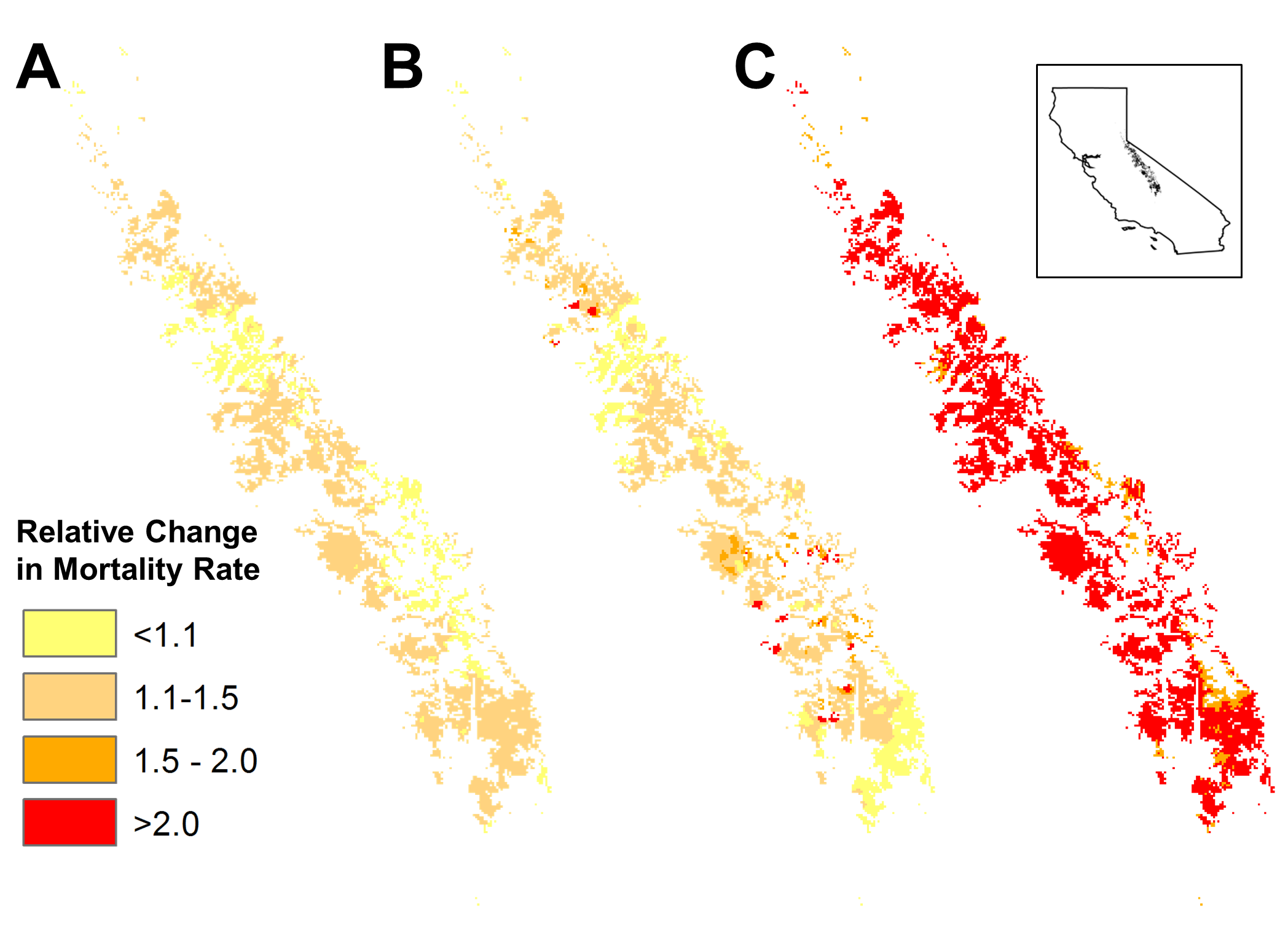

Supplement: Figure S8 — Projected Changes in Mortality Rate for predominantly Energy-Limited Sierra Nevada Conifer Forests (PCM B1, Hypothetical). Mapped projections of average relative changes in mortality rate for the years 2090 to 2099 for energy-limited coniferous forests (≥2450 m) of California’s Sierra Nevada, using exponential models and the PCM B1 emissions model. Elevations generally increase from left to right. (A) Changes in mortality when absolute changes in deficit (Da) are used as a predictor. (B) Changes in mortality when relative changes in deficit (Dr) are used as a predictor. (C) Changes in mortality when absolute changes in temperature (Ta) are used as a predictor. (TIF) [file pone.0069917.s008.tif]
