# Supplementary material for: Climatic Correlates of Tree Mortality in Water- and Energy-Limited Forests
Source: PLoS One. 2013 Jul 25;8(7):e69917. doi: 10.1371/journal.pone.0069917 (PMC3723662; doi:10.1371/journal.pone.0069917)
Supplement: Table S1 — Plot Details. Characteristics of the 21 forest plots used for model development. (DOC) [file pone.0069917.s009.doc]

**Table S1.** **Plot Details.** Characteristics of the 21 forest plots used for model development.

| **Plot** | **Lat** | **Long** | **Elevation** | **Plot area** | **Establishment Year** | **Density** |  |
| --- | --- | --- | --- | --- | --- | --- | --- |
| **Identifier** | **(°N)** | **(°W)** | **(m)** | **(ha)** |  | **(trees/ha)** | **Species comprising > 1% of stems*** |
| YOHOPIPO | 37.8 | 119.9 | 1500 | 1.00 | 1991 | 2980 | ABCO 39%; CADE 32%; PILA 23%; PIPO 5%; PSME 1%; QUKE 1% |
| BBBPIPO | 36.6 | 118.8 | 1609 | 1.00 | 1992 | 1273 | ABCO 12%; CADE 50%; PILA 7%; PIPO 4%; QUCH 4%; QUKE 23% |
| CCRPIPO | 36.6 | 118.8 | 1637 | 1.13 | 1991 | 2101 | ABCO 37%; CADE 34%; PILA 7%; PIPO 6%; QUKE 15% |
| CRCRPIPO | 37.7 | 119.8 | 1637 | 1.00 | 1993 | 1753 | ABCO 46%; CADE 28%; PILA 18%; PIPO 6%; QUKE 2% |
| SURIP | 36.6 | 118.8 | 2033 | 1.38 | 1982 | 743 | ABCO 56%; ABMA 5%; CADE 18%; PILA 16%; QUKE 5% |
| SUABCO | 36.6 | 118.8 | 2035 | 0.88 | 1983 | 773 | ABCO 60%; ABMA 5%; CADE 27%; PILA 8% |
| SUPILA | 36.6 | 118.8 | 2059 | 1.13 | 1982^†^ | 677 | ABCO 66%; CADE 12%; PILA 18%; QUKE 4% |
| FRPIJE | 36.6 | 118.8 | 2106 | 1.00 | 1983 | 177 | ABCO 6%; CADE 3%; PIJE 51%; PILA 1%; QUKE^††^ 37% |
| LMCC | 36.6 | 118.7 | 2128 | 2.00 | 1982 | 336 | ABCO 68%; ABMA 24%; PILA 2%; SEGI 5% |
| LOGSEGI | 36.6 | 118.7 | 2170 | 2.50 | 1983 | 422 | ABCO 76%; ABMA 17%; PILA 4%; SEGI 2% |
| LOLOG | 36.6 | 118.7 | 2207 | 1.13 | 1985 | 405 | ABCO 71%; ABMA 27%; PILA 1%; SEGI 1% |
| UPLOG | 36.6 | 118.7 | 2210 | 1.00 | 1987^†^ | 434 | ABCO 88%; ABMA 1%; CADE 3%; PIJE 1%; PILA 6%; QUKE 1% |
| LOGPIJE | 36.6 | 118.7 | 2405 | 1.00 | 1985 | 121 | ABCO 60%; ABMA 2%; PIJE 33%; PILA 1%; QUKE 4% |
| SFTRABMA | 37.8 | 119.7 | 2484 | 1.00 | 1992 | 1630 | ABMA 100% |
| WTABMA | 36.6 | 118.7 | 2521 | 1.00 | 1993 | 459 | ABMA 99%; PIMO 1% |
| POFLABMA | 37.8 | 119.6 | 2542 | 1.00 | 1994 | 589 | ABMA 95%; PICO 5% |
| PGABMA | 36.6 | 118.7 | 2576 | 1.00 | 1992 | 765 | ABMA 100% |
| EMSLOPE | 36.6 | 118.7 | 2950 | 1.00 | 1983 | 62 | ABMA 1%; PICO 8%; PIJE 8%; PIMO 82% |
| EMRIDGE | 36.6 | 118.7 | 3097 | 1.00 | 1985 | 92 | PICO 6%; PIMO 94% |
| CIRQUE | 36.5 | 118.3 | 3353 | 2.00 | 1993 | 124 | PIBA 99%; PICO 1% |
| GIBBS | 37.9 | 119.2 | 3415 | 2.5 | 1996^†^ | 383 | PIAL 99%; PICO 1% |

* Species composition of stems at time of plot establishment. Percentages may not add to 100 due to rounding. ABCO = *Abies concolor*, ABMA = *A. magnifica*, CADE = *Calocedrus decurrens*, PIAL = *P. albicaulis*, PIBA = *P. balfouriana*, PICO = *P. contorta*, PIJE = *P. jeffreyi*, PILA = *P. lambertiana*, PIMO = *P. monticola*, PIPO = *P. ponderosa*, PIMO = *P. monticola*, PSME = *Pseudotsuga menziesii*, QUCH = *Quercus chrysolepis*, QUKE = *Q. kelloggii*, SEGI = *Sequoiadendron giganteum*. Naming conventions follow Hickman (1993).

† Plots were not completely censused until the following year. Demographic rates were calculated only when the plots were completely censused.
