# Supplementary material for: Climatic Correlates of Tree Mortality in Water- and Energy-Limited Forests
Source: PLoS One. 2013 Jul 25;8(7):e69917. doi: 10.1371/journal.pone.0069917 (PMC3723662; doi:10.1371/journal.pone.0069917)
Supplement: Table S2 — Plot Mortality Data. Counts of mortalities and live trees for each plot for each census year. (DOC) [file pone.0069917.s010.doc]

**Table S2. Plot Mortality Data.** Counts of mortalities and live trees for each plot for each census year.

| **Plot** | **Year** | **# of Mortalities** | **# of Mortalities from Non-Mechanical Causes** | **# of Live Trees from Prior Year*** |
| --- | --- | --- | --- | --- |
| BBBPIPO | 1993 | 68 | 67 | 1273.0 |
| BBBPIPO | 1994 | 23 | 22 | 1217.0 |
| BBBPIPO | 1995 | 28 | 23 | 1206.0 |
| BBBPIPO | 1996 | 11 | 7 | 1190.0 |
| BBBPIPO | 1997 | 13 | 4 | 1191.0 |
| BBBPIPO | 1998 | 14 | 11 | 1191.0 |
| BBBPIPO | 1999 | 3 | 2 | 1189.2 |
| BBBPIPO | 2000 | 16 | 10 | 1198.4 |
| BBBPIPO | 2001 | 7 | 6 | 1194.6 |
| BBBPIPO | 2002 | 16 | 12 | 1199.8 |
| BBBPIPO | 2003 | 16 | 15 | 1198.0 |
| BBBPIPO | 2004 | 17 | 16 | 1193.8 |
| BBBPIPO | 2005 | 9 | 9 | 1188.6 |
| BBBPIPO | 2006 | 10 | 10 | 1191.4 |
| CCRPIPO | 1992 | 37 | 35 | 2101.0 |
| CCRPIPO | 1993 | 56 | 53 | 2119.8 |
| CCRPIPO | 1994 | 66 | 58 | 2119.6 |
| CCRPIPO | 1995 | 30 | 25 | 2109.4 |
| CCRPIPO | 1996 | 45 | 41 | 2135.2 |
| CCRPIPO | 1997 | 28 | 25 | 2148.0 |
| CCRPIPO | 1998 | 41 | 36 | 2206.0 |
| CCRPIPO | 1999 | 25 | 25 | 2251.0 |
| CCRPIPO | 2000 | 42 | 41 | 2312.0 |
| CCRPIPO | 2001 | 46 | 45 | 2356.0 |
| CCRPIPO | 2002 | 64 | 56 | 2397.0 |
| CCRPIPO | 2003 | 49 | 47 | 2377.8 |
| CCRPIPO | 2004 | 35 | 30 | 2373.5 |
| CCRPIPO | 2005 | 30 | 30 | 2383.3 |
| CCRPIPO | 2006 | 59 | 56 | 2399.0 |
| CIRQUE | 1994 | 0 | 0 | 211.0 |
| CIRQUE | 1995 | 0 | 0 | 216.4 |
| CIRQUE | 1996 | 0 | 0 | 221.9 |
| CIRQUE | 1997 | 0 | 0 | 227.3 |
| CIRQUE | 1998 | 0 | 0 | 232.7 |
| CIRQUE | 1999 | 0 | 0 | 238.1 |
| CIRQUE | 2000 | 0 | 0 | 243.6 |
| CIRQUE | 2001 | 0 | 0 | 249.0 |
| CIRQUE | 2002 | 0 | 0 | 251.5 |
| CIRQUE | 2003 | 2 | 2 | 254.0 |
| CIRQUE | 2004 | 0 | 0 | 254.5 |
| CIRQUE | 2005 | 0 | 0 | 253.0 |
| CIRQUE | 2006 | 0 | 0 | 253.0 |
| CRCRPIPO | 1994 | 17 | 15 | 1753.0 |
| CRCRPIPO | 1995 | 21 | 11 | 1748.4 |
| CRCRPIPO | 1996 | 33 | 29 | 1739.8 |
| CRCRPIPO | 1997 | 28 | 18 | 1719.2 |
| CRCRPIPO | 1998 | 22 | 20 | 1703.6 |
| CRCRPIPO | 1999 | 15 | 13 | 1694.0 |
| CRCRPIPO | 2000 | 21 | 19 | 1696.2 |
| CRCRPIPO | 2001 | 26 | 22 | 1692.4 |
| CRCRPIPO | 2002 | 41 | 37 | 1683.6 |
| CRCRPIPO | 2003 | 37 | 30 | 1659.8 |
| CRCRPIPO | 2004 | 34 | 29 | 1640.0 |
| CRCRPIPO | 2005 | 26 | 24 | 1606.0 |
| CRCRPIPO | 2006 | 18 | 18 | 1580.0 |
| EMRIDGE | 1986 | 0 | 0 | 92.0 |
| EMRIDGE | 1987 | 0 | 0 | 92.3 |
| EMRIDGE | 1988 | 0 | 0 | 92.5 |
| EMRIDGE | 1989 | 0 | 0 | 92.8 |
| EMRIDGE | 1990 | 2 | 2 | 93.0 |
| EMRIDGE | 1991 | 2 | 2 | 92.2 |
| EMRIDGE | 1992 | 0 | 0 | 91.4 |
| EMRIDGE | 1993 | 0 | 0 | 92.6 |
| EMRIDGE | 1994 | 1 | 0 | 93.8 |
| EMRIDGE | 1995 | 0 | 0 | 94.0 |
| EMRIDGE | 1996 | 0 | 0 | 96.0 |
| EMRIDGE | 1997 | 0 | 0 | 98.0 |
| EMRIDGE | 1998 | 0 | 0 | 100.0 |
| EMRIDGE | 1999 | 0 | 0 | 102.0 |
| EMRIDGE | 2000 | 0 | 0 | 104.0 |
| EMRIDGE | 2001 | 0 | 0 | 106.3 |
| EMRIDGE | 2002 | 0 | 0 | 108.5 |
| EMRIDGE | 2003 | 1 | 1 | 110.8 |
| EMRIDGE | 2004 | 0 | 0 | 112.0 |
| EMRIDGE | 2005 | 0 | 0 | 112.0 |
| EMRIDGE | 2006 | 0 | 0 | 112.0 |
| EMSLOPE | 1984 | 0 | 0 | 62.0 |
| EMSLOPE | 1985 | 2 | 2 | 62.2 |
| EMSLOPE | 1986 | 0 | 0 | 60.4 |
| EMSLOPE | 1987 | 0 | 0 | 60.6 |
| EMSLOPE | 1988 | 1 | 1 | 60.8 |
| EMSLOPE | 1989 | 1 | 1 | 60.0 |
| EMSLOPE | 1990 | 0 | 0 | 59.4 |
| EMSLOPE | 1991 | 0 | 0 | 59.8 |
| EMSLOPE | 1992 | 0 | 0 | 60.2 |
| EMSLOPE | 1993 | 0 | 0 | 60.6 |
| EMSLOPE | 1994 | 0 | 0 | 61.0 |
| EMSLOPE | 1995 | 0 | 0 | 61.4 |
| EMSLOPE | 1996 | 0 | 0 | 61.8 |
| EMSLOPE | 1997 | 1 | 1 | 62.2 |
| EMSLOPE | 1998 | 0 | 0 | 61.6 |
| EMSLOPE | 1999 | 0 | 0 | 62.0 |
| EMSLOPE | 2000 | 1 | 0 | 62.4 |
| EMSLOPE | 2001 | 0 | 0 | 61.8 |
| EMSLOPE | 2002 | 0 | 0 | 62.2 |
| EMSLOPE | 2003 | 0 | 0 | 62.6 |
| EMSLOPE | 2004 | 0 | 0 | 63.0 |
| EMSLOPE | 2005 | 0 | 0 | 63.0 |
| EMSLOPE | 2006 | 0 | 0 | 63.0 |
| FRPIJE | 1984 | 4 | 4 | 161.0 |
| FRPIJE | 1985 | 0 | 0 | 158.0 |
| FRPIJE | 1986 | 5 | 5 | 159.0 |
| FRPIJE | 1987 | 0 | 0 | 155.0 |
| FRPIJE | 1988 | 2 | 1 | 156.0 |
| FRPIJE | 1989 | 5 | 5 | 155.0 |
| FRPIJE | 1990 | 1 | 1 | 152.0 |
| FRPIJE | 1991 | 2 | 2 | 153.0 |
| FRPIJE | 1992 | 2 | 2 | 153.0 |
| FRPIJE | 1993 | 1 | 1 | 153.0 |
| FRPIJE | 1994 | 6 | 6 | 154.0 |
| FRPIJE | 1995 | 2 | 2 | 150.0 |
| FRPIJE | 1996 | 8 | 6 | 150.8 |
| FRPIJE | 1997 | 3 | 3 | 145.6 |
| FRPIJE | 1998 | 5 | 2 | 145.4 |
| FRPIJE | 1999 | 7 | 6 | 143.2 |
| FRPIJE | 2000 | 2 | 0 | 139.0 |
| FRPIJE | 2001 | 1 | 1 | 140.3 |
| FRPIJE | 2002 | 0 | 0 | 142.5 |
| FRPIJE | 2003 | 4 | 4 | 145.8 |
| FRPIJE | 2004 | 11 | 11 | 145.0 |
| FRPIJE | 2005 | 3 | 3 | 134.0 |
| FRPIJE | 2006 | 6 | 6 | 131.0 |
| GIBBS | 1998 | 12 | 10 | 973.0 |
| GIBBS | 1999 | 2 | 1 | 961.0 |
| GIBBS | 2000 | 8 | 1 | 960.0 |
| GIBBS | 2001 | 6 | 6 | 965.0 |
| GIBBS | 2002 | 8 | 7 | 962.0 |
| GIBBS | 2003 | 12 | 10 | 964.0 |
| GIBBS | 2004 | 5 | 5 | 952.0 |
| GIBBS | 2005 | 7 | 7 | 949.0 |
| GIBBS | 2006 | 6 | 4 | 942.0 |
| LMCC | 1983 | 8 | 0 | 591.0 |
| LMCC | 1984 | 11 | 3 | 591.2 |
| LMCC | 1985 | 8 | 3 | 588.3 |
| LMCC | 1986 | 5 | 1 | 588.5 |
| LMCC | 1987 | 3 | 1 | 591.7 |
| LMCC | 1988 | 8 | 6 | 596.8 |
| LMCC | 1989 | 9 | 9 | 597.0 |
| LMCC | 1990 | 6 | 6 | 593.0 |
| LMCC | 1991 | 6 | 6 | 592.0 |
| LMCC | 1992 | 7 | 7 | 591.0 |
| LMCC | 1993 | 5 | 4 | 589.0 |
| LMCC | 1994 | 8 | 7 | 589.0 |
| LMCC | 1995 | 5 | 4 | 586.0 |
| LMCC | 1996 | 7 | 5 | 594.8 |
| LMCC | 1997 | 9 | 6 | 601.6 |
| LMCC | 1998 | 7 | 5 | 606.4 |
| LMCC | 1999 | 8 | 3 | 613.2 |
| LMCC | 2000 | 13 | 12 | 620.0 |
| LMCC | 2001 | 4 | 4 | 615.5 |
| LMCC | 2002 | 11 | 10 | 620.0 |
| LMCC | 2003 | 6 | 4 | 617.5 |
| LMCC | 2004 | 13 | 13 | 620.0 |
| LMCC | 2005 | 3 | 3 | 607.0 |
| LMCC | 2006 | 8 | 8 | 604.0 |
| LOGPIJE | 1986 | 0 | 0 | 119.0 |
| LOGPIJE | 1987 | 1 | 1 | 120.2 |
| LOGPIJE | 1988 | 0 | 0 | 120.4 |
| LOGPIJE | 1989 | 0 | 0 | 121.6 |
| LOGPIJE | 1990 | 1 | 1 | 122.8 |
| LOGPIJE | 1991 | 1 | 1 | 123.0 |
| LOGPIJE | 1992 | 2 | 2 | 125.0 |
| LOGPIJE | 1993 | 3 | 3 | 126.0 |
| LOGPIJE | 1994 | 2 | 2 | 126.0 |
| LOGPIJE | 1995 | 0 | 0 | 127.0 |
| LOGPIJE | 1996 | 1 | 1 | 130.0 |
| LOGPIJE | 1997 | 5 | 3 | 132.6 |
| LOGPIJE | 1998 | 0 | 0 | 131.2 |
| LOGPIJE | 1999 | 1 | 1 | 134.8 |
| LOGPIJE | 2000 | 2 | 0 | 137.4 |
| LOGPIJE | 2001 | 2 | 2 | 139.0 |
| LOGPIJE | 2002 | 1 | 1 | 138.6 |
| LOGPIJE | 2003 | 3 | 3 | 139.2 |
| LOGPIJE | 2004 | 1 | 1 | 137.8 |
| LOGPIJE | 2005 | 2 | 2 | 138.4 |
| LOGPIJE | 2006 | 5 | 5 | 138.0 |
| LOGSEGI | 1984 | 5 | 4 | 1055.0 |
| LOGSEGI | 1985 | 5 | 5 | 1067.0 |
| LOGSEGI | 1986 | 8 | 7 | 1079.0 |
| LOGSEGI | 1987 | 13 | 11 | 1088.0 |
| LOGSEGI | 1988 | 5 | 4 | 1092.0 |
| LOGSEGI | 1989 | 18 | 16 | 1105.0 |
| LOGSEGI | 1990 | 12 | 7 | 1096.7 |
| LOGSEGI | 1991 | 25 | 20 | 1094.3 |
| LOGSEGI | 1992 | 9 | 8 | 1079.0 |
| LOGSEGI | 1993 | 8 | 7 | 1079.7 |
| LOGSEGI | 1994 | 8 | 8 | 1081.3 |
| LOGSEGI | 1995 | 8 | 5 | 1083.0 |
| LOGSEGI | 1996 | 9 | 4 | 1091.8 |
| LOGSEGI | 1997 | 8 | 6 | 1099.6 |
| LOGSEGI | 1998 | 16 | 13 | 1108.4 |
| LOGSEGI | 1999 | 11 | 8 | 1109.2 |
| LOGSEGI | 2000 | 16 | 13 | 1118.0 |
| LOGSEGI | 2001 | 11 | 10 | 1113.2 |
| LOGSEGI | 2002 | 15 | 14 | 1113.4 |
| LOGSEGI | 2003 | 13 | 13 | 1109.6 |
| LOGSEGI | 2004 | 18 | 18 | 1107.8 |
| LOGSEGI | 2005 | 8 | 8 | 1101.0 |
| LOGSEGI | 2006 | 9 | 9 | 1093.0 |
| LOLOG | 1988 | 0 | 0 | 451.0 |
| LOLOG | 1989 | 4 | 4 | 454.4 |
| LOLOG | 1990 | 10 | 10 | 453.8 |
| LOLOG | 1991 | 14 | 14 | 447.2 |
| LOLOG | 1992 | 9 | 6 | 436.6 |
| LOLOG | 1993 | 5 | 5 | 428.0 |
| LOLOG | 1994 | 6 | 3 | 431.4 |
| LOLOG | 1995 | 1 | 0 | 433.8 |
| LOLOG | 1996 | 6 | 4 | 441.2 |
| LOLOG | 1997 | 6 | 2 | 443.6 |
| LOLOG | 1998 | 5 | 4 | 447.0 |
| LOLOG | 1999 | 3 | 1 | 446.0 |
| LOLOG | 2000 | 3 | 0 | 447.0 |
| LOLOG | 2001 | 4 | 4 | 448.0 |
| LOLOG | 2002 | 3 | 3 | 448.0 |
| LOLOG | 2003 | 9 | 7 | 449.0 |
| LOLOG | 2004 | 2 | 1 | 442.4 |
| LOLOG | 2005 | 6 | 4 | 442.8 |
| LOLOG | 2006 | 8 | 5 | 439.2 |
| PGABMA | 1993 | 0 | 0 | 765.0 |
| PGABMA | 1994 | 14 | 11 | 767.0 |
| PGABMA | 1995 | 7 | 4 | 755.0 |
| PGABMA | 1996 | 13 | 6 | 750.0 |
| PGABMA | 1997 | 11 | 6 | 739.0 |
| PGABMA | 1998 | 13 | 9 | 730.0 |
| PGABMA | 1999 | 10 | 3 | 720.4 |
| PGABMA | 2000 | 18 | 4 | 713.8 |
| PGABMA | 2001 | 12 | 3 | 699.2 |
| PGABMA | 2002 | 10 | 3 | 690.6 |
| PGABMA | 2003 | 13 | 8 | 684.0 |
| PGABMA | 2004 | 13 | 9 | 671.8 |
| PGABMA | 2005 | 8 | 8 | 659.6 |
| PGABMA | 2006 | 13 | 9 | 652.4 |
| POFLABMA | 1995 | 1 | 0 | 589.0 |
| POFLABMA | 1996 | 2 | 1 | 594.8 |
| POFLABMA | 1997 | 5 | 2 | 599.6 |
| POFLABMA | 1998 | 7 | 5 | 601.4 |
| POFLABMA | 1999 | 8 | 6 | 601.2 |
| POFLABMA | 2000 | 6 | 6 | 600.0 |
| POFLABMA | 2001 | 5 | 4 | 606.4 |
| POFLABMA | 2002 | 4 | 4 | 613.8 |
| POFLABMA | 2003 | 1 | 1 | 622.2 |
| POFLABMA | 2004 | 9 | 8 | 634.6 |
| POFLABMA | 2005 | 5 | 3 | 637.0 |
| POFLABMA | 2006 | 3 | 3 | 632.0 |
| SFTRABMA | 1993 | 12 | 9 | 1631.0 |
| SFTRABMA | 1994 | 12 | 10 | 1623.2 |
| SFTRABMA | 1995 | 10 | 10 | 1615.4 |
| SFTRABMA | 1996 | 28 | 25 | 1609.6 |
| SFTRABMA | 1997 | 24 | 22 | 1585.8 |
| SFTRABMA | 1998 | 19 | 19 | 1566.0 |
| SFTRABMA | 1999 | 18 | 16 | 1559.4 |
| SFTRABMA | 2000 | 41 | 39 | 1553.8 |
| SFTRABMA | 2001 | 39 | 37 | 1525.2 |
| SFTRABMA | 2002 | 24 | 24 | 1498.6 |
| SFTRABMA | 2003 | 43 | 42 | 1487.0 |
| SFTRABMA | 2004 | 35 | 35 | 1449.0 |
| SFTRABMA | 2005 | 25 | 23 | 1419.0 |
| SFTRABMA | 2006 | 16 | 14 | 1399.0 |
| SUABCO | 1984 | 11 | 8 | 680.0 |
| SUABCO | 1985 | 6 | 6 | 672.6 |
| SUABCO | 1986 | 9 | 8 | 670.2 |
| SUABCO | 1987 | 6 | 6 | 664.8 |
| SUABCO | 1988 | 6 | 4 | 662.4 |
| SUABCO | 1989 | 24 | 23 | 664.0 |
| SUABCO | 1990 | 15 | 12 | 643.2 |
| SUABCO | 1991 | 24 | 23 | 631.3 |
| SUABCO | 1992 | 5 | 5 | 610.5 |
| SUABCO | 1993 | 5 | 5 | 608.7 |
| SUABCO | 1994 | 6 | 6 | 606.8 |
| SUABCO | 1995 | 6 | 5 | 604.0 |
| SUABCO | 1996 | 9 | 5 | 602.6 |
| SUABCO | 1997 | 19 | 11 | 598.2 |
| SUABCO | 1998 | 13 | 9 | 583.8 |
| SUABCO | 1999 | 8 | 7 | 575.4 |
| SUABCO | 2000 | 9 | 8 | 572.0 |
| SUABCO | 2001 | 8 | 8 | 567.4 |
| SUABCO | 2002 | 12 | 11 | 563.8 |
| SUABCO | 2003 | 10 | 10 | 556.2 |
| SUABCO | 2004 | 9 | 9 | 550.6 |
| SUABCO | 2005 | 2 | 1 | 546.0 |
| SUABCO | 2006 | 7 | 4 | 544.0 |
| SUPILA | 1984 | 4 | 4 | 765.0 |
| SUPILA | 1985 | 2 | 2 | 768.2 |
| SUPILA | 1986 | 3 | 2 | 773.4 |
| SUPILA | 1987 | 4 | 4 | 777.6 |
| SUPILA | 1988 | 7 | 6 | 780.8 |
| SUPILA | 1989 | 14 | 13 | 785.0 |
| SUPILA | 1990 | 15 | 15 | 781.5 |
| SUPILA | 1991 | 24 | 24 | 777.0 |
| SUPILA | 1992 | 11 | 10 | 763.5 |
| SUPILA | 1993 | 3 | 3 | 763.0 |
| SUPILA | 1994 | 11 | 11 | 770.5 |
| SUPILA | 1995 | 7 | 6 | 770.0 |
| SUPILA | 1996 | 16 | 15 | 778.6 |
| SUPILA | 1997 | 10 | 8 | 778.2 |
| SUPILA | 1998 | 9 | 6 | 783.8 |
| SUPILA | 1999 | 3 | 2 | 790.4 |
| SUPILA | 2000 | 18 | 17 | 804.0 |
| SUPILA | 2001 | 10 | 9 | 796.4 |
| SUPILA | 2002 | 7 | 7 | 796.8 |
| SUPILA | 2003 | 19 | 19 | 800.2 |
| SUPILA | 2004 | 22 | 22 | 791.6 |
| SUPILA | 2005 | 7 | 6 | 780.0 |
| SUPILA | 2006 | 20 | 19 | 773.0 |
| SURIP | 1984 | 7 | 5 | 1014.0 |
| SURIP | 1985 | 6 | 6 | 1020.4 |
| SURIP | 1986 | 7 | 7 | 1027.8 |
| SURIP | 1987 | 9 | 7 | 1034.2 |
| SURIP | 1988 | 6 | 5 | 1038.6 |
| SURIP | 1989 | 16 | 16 | 1048.0 |
| SURIP | 1990 | 12 | 9 | 1041.7 |
| SURIP | 1991 | 24 | 22 | 1039.3 |
| SURIP | 1992 | 13 | 12 | 1025.0 |
| SURIP | 1993 | 3 | 3 | 1021.7 |
| SURIP | 1994 | 23 | 21 | 1028.3 |
| SURIP | 1995 | 19 | 10 | 1015.0 |
| SURIP | 1996 | 24 | 20 | 1014.0 |
| SURIP | 1997 | 13 | 9 | 1008.0 |
| SURIP | 1998 | 30 | 17 | 1013.0 |
| SURIP | 1999 | 15 | 10 | 1001.0 |
| SURIP | 2000 | 32 | 29 | 1006.0 |
| SURIP | 2001 | 13 | 12 | 984.8 |
| SURIP | 2002 | 13 | 9 | 982.6 |
| SURIP | 2003 | 11 | 8 | 980.4 |
| SURIP | 2004 | 16 | 15 | 980.2 |
| SURIP | 2005 | 1 | 1 | 975.0 |
| SURIP | 2006 | 25 | 21 | 974.0 |
| UPLOG | 1989 | 1 | 1 | 407.0 |
| UPLOG | 1990 | 2 | 2 | 409.5 |
| UPLOG | 1991 | 4 | 4 | 411.0 |
| UPLOG | 1992 | 2 | 2 | 410.5 |
| UPLOG | 1993 | 5 | 5 | 412.0 |
| UPLOG | 1994 | 2 | 2 | 413.2 |
| UPLOG | 1995 | 12 | 1 | 417.4 |
| UPLOG | 1996 | 4 | 2 | 411.6 |
| UPLOG | 1997 | 6 | 3 | 413.8 |
| UPLOG | 1998 | 3 | 2 | 416.0 |
| UPLOG | 1999 | 4 | 3 | 415.2 |
| UPLOG | 2000 | 2 | 1 | 413.4 |
| UPLOG | 2001 | 1 | 1 | 413.6 |
| UPLOG | 2002 | 6 | 5 | 414.8 |
| UPLOG | 2003 | 6 | 4 | 412.0 |
| UPLOG | 2004 | 2 | 2 | 407.8 |
| UPLOG | 2005 | 5 | 1 | 407.6 |
| UPLOG | 2006 | 2 | 2 | 404.4 |
| WTABMA | 1994 | 1 | 1 | 459.0 |
| WTABMA | 1995 | 7 | 3 | 460.8 |
| WTABMA | 1996 | 1 | 0 | 456.6 |
| WTABMA | 1997 | 5 | 2 | 458.4 |
| WTABMA | 1998 | 0 | 0 | 456.2 |
| WTABMA | 1999 | 1 | 0 | 460.0 |
| WTABMA | 2000 | 8 | 7 | 461.6 |
| WTABMA | 2001 | 1 | 0 | 456.2 |
| WTABMA | 2002 | 7 | 4 | 457.8 |
| WTABMA | 2003 | 10 | 3 | 453.4 |
| WTABMA | 2004 | 9 | 8 | 446.0 |
| WTABMA | 2005 | 7 | 6 | 437.0 |
| WTABMA | 2006 | 6 | 5 | 430.0 |
| YOHOPIPO | 1992 | 44 | 44 | 2965.0 |
| YOHOPIPO | 1993 | 44 | 43 | 2959.5 |
| YOHOPIPO | 1994 | 22 | 22 | 2954.0 |
| YOHOPIPO | 1995 | 40 | 34 | 2970.5 |
| YOHOPIPO | 1996 | 52 | 45 | 2969.0 |
| YOHOPIPO | 1997 | 39 | 34 | 2955.5 |
| YOHOPIPO | 1998 | 58 | 54 | 2956.0 |
| YOHOPIPO | 1999 | 67 | 61 | 2966.8 |
| YOHOPIPO | 2000 | 43 | 41 | 2968.5 |
| YOHOPIPO | 2001 | 71 | 70 | 2994.3 |
| YOHOPIPO | 2002 | 122 | 117 | 2993.0 |
| YOHOPIPO | 2003 | 130 | 116 | 2904.2 |
| YOHOPIPO | 2004 | 76 | 66 | 2807.4 |
| YOHOPIPO | 2005 | 89 | 88 | 2764.6 |
| YOHOPIPO | 2006 | 32 | 32 | 2709.8 |

* For most plots for certain time periods, new recruitment was only measured at the time of DBH measurements (usually every 5 years). For purposes of the analysis, recruitment was distributed evenly throughout the interval, resulting at times in ‘fractional’ trees.
